# Tumor Immune Contexture Model Predicts Prognosis and Immunotherapy Response in Gastric Cancer

**DOI:** 10.1002/jgm.70065

**Published:** 2025-12-17

**Authors:** Jiexuan Wang, Xin Yang, Xuan Dai, Zhiqian Hu, Xinxing Li

**Affiliations:** ^1^ Department of General Surgery, Tongji Hospital, School of Medicine Tongji University Shanghai China

**Keywords:** gastric cancer, immune microenvironment, immunotherapy, machine learning, prognostic model

## Abstract

**Background:**

Gastric cancer (GC) is highly heterogeneous, and current prognostic models fail to fully capture tumor immune characteristics, limiting personalized treatment. This study introduces the Tumor Immune Environment Score (TIES), an immune‐based prognostic model designed to enhance risk stratification and predict response to immunotherapy.

**Methods:**

Transcriptomic and clinical data from seven GC cohorts, comprising a total of 1487 patients, were analyzed. The training cohort included GSE15459, GSE62254, GSE84433, and GSE13861, while the validation cohort comprised GSE26899, GSE26901, and TCGA‐STAD. Immune‐related gene signatures were quantified using ssGSEA and subsequently analyzed via LASSO and Cox regression. Clinical applicability was quantified using decision‐curve analysis (DCA) and reclassification metrics (category‐free NRI/IDI) for TIES versus TIES + pathological stage. Immunotherapy response was assessed using data from GSE183924, and its associations with immune characteristics, molecular alterations, and drug sensitivity were investigated. For experimental confirmation, we established a 12‐gene RT‐qPCR panel in 30 institutional GC specimens and computed a weighted qPCR surrogate (qTIES).

**Results:**

A high TIES was associated with worse OS (HR = 2.44, *p* < 0.001) and DFS (HR = 2.25, *p* < 0.001). TIES was an independent prognostic factor and demonstrated superior predictive accuracy compared to TNM staging (AUC = 0.75–0.79 vs. 0.61–0.64, *p* < 0.001). Integrating clinical‐pathological features with TIES significantly enhanced predictive performance (AUC = 0.75–0.78 vs. 0.61–0.63, *p* < 0.01). A low TIES was indicative of an immune‐inflamed subtype, characterized by a higher TMB (*p* < 0.01) and a greater likelihood of response to immunotherapy (HR = 0.24, *p* = 0.014). TIES outperformed TIDE in predicting immunotherapy outcomes, achieving a higher OS C‐index (0.780 vs. 0.743) and DFS C‐index (0.664 vs. 0.610). At 3 years, TIES + stage yielded a greater net benefit than TIES alone across clinically relevant thresholds (DCA) and improved NRI/IDI.The qPCR panel reproduced axis biology (CSR/TGF‐βincreased with NK/Th2 decreased), correlated with pathological stage, stratified OS/DFS by Kaplan–Meier, and showed 3‐year discrimination.

**Conclusion:**

TIES represents a robust immune‐based prognostic tool for GC, facilitating improved risk stratification and immunotherapy response prediction. Its integration into clinical practice, particularly in combination with clinical‐pathological features, may enhance personalized treatment strategies and improve patient outcomes.

## Graphical abstract

We developed the Tumor Immune Environment Score (TIES), an immune–stromal model derived from 1487 gastric cancers across seven cohorts. TIES integrates four axes—cancer–stroma remodeling, NK‐cell activity, TGF‐β signaling, and Th2 polarization—to stratify patients into high‐ and low‐risk groups. Low TIES tumors show immune‐inflamed features, higher TMB and MSI, and improved survival and response to PD‐L1 blockade compared with high TIES tumors. A 12‐gene RT‐qPCR panel (qTIES) recapitulates TIES biology and enables practical risk stratification in clinical samples, supporting immune contexture–guided precision management of gastric cancer.

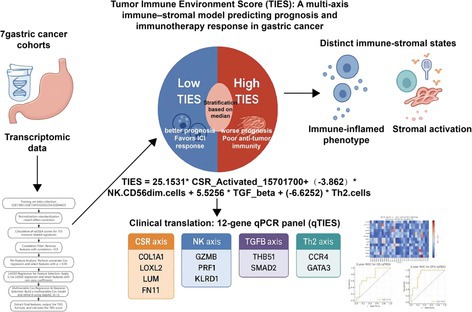

**Abbreviations**
AUC: area under the curve
AJCC: American Joint Committee on Cancer
CAF: cancer‐associated fibroblast
C‐index: concordance index
CPS: combined positive score
DFS: disease‐free survival
EMT: epithelial‐mesenchymal transition
ESTIMATE: Estimation of STromal and Immune cells in MAlignant Tumor tissues using Expression data
GC: gastric cancer
GDSC: genomics of drug sensitivity in cancer
GEO: gene expression omnibus
GSEA: gene set enrichment analysis
HR: hazard ratio
HRD: homologous recombination deficiency
IC50: half‐maximal inhibitory concentration
ICIs: immune checkpoint inhibitors
IFN‐γ: interferon gamma
LASSO: least absolute shrinkage and selection operator
LOH: loss of heterozygosity
LST: large‐scale state transition
MSI‐H: microsatellite instability‐high
NK: natural killer
NtAI: telomeric allelic imbalance
OS: overall survival
pStage: pathological stage
PD‐1: programmed death‐1
PD‐L1: programmed death‐ligand 1
TCGA: The Cancer Genome Atlas
TAM: tumor‐associated macrophage
TIDE: tumor immune dysfunction and exclusion
TIES: tumor immune environment score
TIS: T‐cell inflammation signatures
TMB: tumor mutational burden
TME: tumor microenvironment
TNM: tumor
Node: metastasis
ssGSEA: single‐sample gene set enrichment analysis

## 1 Introduction

Gastric cancer (GC) continues to pose a major global health burden, with over one million new cases diagnosed in 2020, making it the fifth most prevalent malignancy worldwide [1]. Despite significant progress in multimodal therapeutic approaches, the prognosis remains unfavorable, with a five‐year overall survival (OS) rate of only 25%–30% [2]. Despite advances in modern therapeutic approaches, their impact on long‐term survival remains limited [3], highlighting the urgent need for improved prognostic stratification and personalized treatment strategies.

In clinical settings, prognostic assessment frequently relies on tumor staging systems like the TNM classification, along with serum tumor markers such as carcinoembryonic antigen (CEA) and carbohydrate antigen 19–9 (CA19–9) [4]. However, these markers fail to capture the biological heterogeneity of GC. Patients with similar pathological stages or clinical characteristics often exhibit markedly different survival outcomes, suggesting that conventional risk factors alone are insufficient for precise prognostication [5]. Consequently, identifying novel molecular biomarkers is essential for improving risk stratification and guiding individualized therapeutic decisions.

Recent advances in GC treatment have emphasized immunotherapy, with PD‐1/PD‐L1‐targeting immune checkpoint blockade emerging as a standard strategy for managing advanced disease [6]. Although ICIs can induce durable responses in some patients, their overall objective response rate remains below 50%, with variability observed across different studies and patient populations [7]. Reliable predictive biomarkers for immunotherapy response beyond microsatellite instability‐high (MSI‐H) remain scarce [8]. The prognostic significance of PD‐L1 expression, commonly evaluated through the combined positive score (CPS), remains debated [9]. However, it continues to serve as a crucial biomarker in certain clinical contexts. Similarly, tumor mutational burden (TMB) has yet to be universally validated as a definitive predictor of immunotherapy response in GC, although high TMB may still be associated with improved responses in certain cases [10]. This variability in treatment efficacy underscores the need for additional prognostic indicators linked to tumor immunity to optimize patient selection for immunotherapy.

In recent years, immune‐related factors have been increasingly recognized as key determinants of cancer progression and patient survival. Growing evidence indicates that tumor‐infiltrating lymphocytes (TILs) and other immune elements within the tumor microenvironment (TME) play a crucial role in shaping tumor behavior and prognosis across various malignancies [11]. The TME comprises cancer cells, immune cells, stromal components, and cytokines, all of which influence tumor progression and therapeutic response [6]. Certain lymphocyte subpopulations have been linked to improved survival outcomes in specific cancers [12], whereas the presence of immunosuppressive cells, such as M2 macrophages and regulatory T cells, can facilitate tumor progression and contribute to treatment resistance [13]. These findings highlight the necessity of integrating tumor immune landscape analysis into prognostic evaluation for gastric cancer (GC).

Given the pivotal role of tumor‐immune interactions, numerous prognostic gene expression signatures enriched in immune‐related genes have been proposed [14, 15]. These immune‐based signatures have the potential to complement traditional staging systems by refining risk stratification, though their reproducibility and clinical utility require further validation. However, many existing GC prognostic models are derived from single‐cohort studies or lack external validation, raising concerns regarding their generalizability. Moreover, few studies have systematically evaluated gene expression‐based prognostic models in direct comparison with established immunotherapy biomarkers, such as the Tumor Immune Dysfunction and Exclusion (TIDE) metric and T‐cell inflammation signatures (TIS), leading to uncertainty regarding their clinical applicability [7]. These challenges underscore the necessity for a more refined and comprehensive prognostic framework.

To address these limitations, this study introduces a novel immune‐related prognostic scoring system for GC, designed to enhance risk stratification and guide personalized treatment strategies. By utilizing large‐scale transcriptomic data alongside corresponding clinical outcomes, we identified a distinct set of immune‐related signatures with significant prognostic value and developed the Tumor Immune Environment Score (TIES) as a new risk stratification tool. This model underwent rigorous validation in an independent patient cohort, demonstrating its predictive capability for both overall and disease‐free survival (DFS). Additionally, we explored the relationship between risk groups classified by our model and key tumor‐immune characteristics, such as immune cell infiltration patterns, immune checkpoint activity, and tumor mutational burden (TMB), to better understand the biological underpinnings of prognostic variability. Our immune‐based prognostic signature provides valuable insights into tumor‐immune interactions in GC. By identifying high‐risk patient subgroups and potential immunotherapy responders, this model improves risk stratification and informs personalized treatment strategies for GC. Consequently, our findings contribute to refining prognostic accuracy and optimizing therapeutic decision‐making in gastric cancer management.

## 2 Methods

### 2.1 Collection and Preprocessing of Gastric Cancer Datasets

Transcriptomic profiles and associated clinical and pathological data were collected from seven gastric cancer datasets available in the TCGA and GEO databases, including GSE15459 [16], GSE62254 [17], GSE84433 [18], GSE13861 [19], GSE26899 [20], GSE26901 [20], and TCGA‐STAD [21]. Microarray data from the Affymetrix platform (GSE15459 and GSE62254) underwent normalization using the robust multi‐array average (RMA) method. Meanwhile, datasets generated from the Illumina platform (GSE84433, GSE13861, GSE26899, and GSE26901) were processed through quantile normalization followed by log2 transformation. For RNA‐seq data from TCGA‐STAD, raw counts were converted to transcripts per million (TPM) and subsequently log2(x + 1) transformed. Samples with over 20% missing values in either genes (rows) or samples (columns) were removed. Features with missing values below 20% were imputed using the k‐nearest neighbors (KNN) algorithm. Patients without available prognostic data were excluded from further analysis. The seven datasets were divided into training and test cohorts according to sequencing platforms and sample sizes (see Table S1). To evaluate the predictive power of the TIES model for immunotherapy response, we integrated an independent external dataset, GSE183924, comprising 37 patients treated with durvalumab (anti‐PD‐L1) after gastric cancer surgery [22].

A total of 1487 gastric cancer patients were included in this study, with their comprehensive baseline clinical characteristics detailed in Table S1. Probe identifiers from microarray datasets were mapped to gene symbols. If a probe corresponded to multiple genes, only the first match was retained. Probes without gene annotations were excluded, and for genes associated with multiple probes, the average expression value was calculated. To mitigate batch effects in the training cohort, the “ComBat” algorithm implemented in the “sva” package was applied, accounting for potential biological covariates.

### 2.2 Identification of Prognostic Signatures and Construction of the TIES Model

A systematic literature review identified 155 immune‐related gene signatures from previous studies and publicly available databases (Table S2) [23, 24, 25, 26]. Single‐sample gene set enrichment analysis (ssGSEA) scores were derived from log2‐transformed, normalized expression data in the training cohort. To ensure model stability and reduce redundancy, signatures with a Spearman correlation coefficient > 0.9 were excluded. Univariate Cox proportional hazards regression was applied to the remaining signatures, retaining those with a *p*‐value < 0.05 for further evaluation. LASSO regression, along with multivariate Cox regression and stepwise selection, was employed to refine the predictive model. The final TIES model was determined using the Akaike information criterion (AIC) for model selection. The TIES score was computed as a weighted aggregate of the selected signatures' ssGSEA scores, with weights derived from the corresponding coefficients in the multivariate Cox regression model. To ensure comparability, the final TIES score was standardized using z‐score normalization. Based on the median TIES score, patients were categorized into high‐ and low‐risk groups.

The predictive utility of TIES was assessed using independent test cohorts through multiple validation approaches, including survival analysis and concordance index estimation. The robustness and independence of TIES in predicting survival outcomes were evaluated through subgroup analyses and both univariate and multivariate Cox regression models. Two multivariate regression models were developed to assess the additional prognostic relevance of TIES. The first model incorporated pStage, Lauren classification, and TIES as continuous variables, while the second model included the same set of variables but excluded TIES.

### 2.3 Characterization of Biological Features Associated With TIES

Using TCGA‐STAD data, we analyzed somatic mutation profiles in TIES subgroups, categorized as high and low, and assessed microsatellite instability (MSI), tumor mutation burden (TMB), and homologous recombination deficiency (HRD) scores. Gene set enrichment analysis (GSEA) was performed utilizing Kyoto Encyclopedia of Genes and Genomes (KEGG) pathways from the Molecular Signatures Database (MSigDB) to identify functional distinctions between the two TIES groups. Additionally, oncogenic pathway alterations were examined through ssGSEA using predefined oncogenic gene sets from Sanchez‐Vega et al. [27].

### 2.4 Prediction of Drug Response in High‐ and low‐TIES Groups

The “oncoPredict” package was utilized to quantify the IC50 (half‐maximal inhibitory concentration) of anticancer drugs using tumor expression profiles derived from the Genomics of Drug Sensitivity in Cancer (GDSC) database [28]. The Tumor Immune Dysfunction and Exclusion (TIDE) algorithm was applied to evaluate the prognostic significance of TIES in predicting immunotherapy response [29]. A lower TIDE score suggests a higher likelihood of a positive immunotherapy outcome.

### 2.5 Patient Specimens and Ethical Approval

Thirty primary gastric cancer specimens were collected at Tongji University Affiliated Tongji Hospital between 2017 and 2019. All participants provided written informed consent prior to enrollment. The study protocol was approved by the institutional review board (Ethics approval No.: [ChiECRCT20170153]), in accordance with the Declaration of Helsinki and institutional guidelines. Tumor tissue was processed immediately after resection, snap‐frozen, and stored at −80°C. Clinical variables—including age, sex, pT, pN, pM, and AJCC pathological stage—were abstracted from medical records by two independent investigators. Patients were followed for at least 5 years; overall survival (OS) and disease‐free survival (DFS) were defined by standard criteria (Patient information is as follows: Table S8).

### 2.6 Tissue Processing, RNA Extraction, and cDNA Synthesis

Total RNA was isolated from frozen tumor using silica‐membrane spin columns with on‐column DNase digestion. RNA integrity and purity were confirmed spectrophotometrically and by agarose electrophoresis. cDNA was synthesized from 1 μg RNA using a reverse‐transcription kit with random hexamers according to the manufacturer's instructions.

### 2.7 RT‐qPCR Assay and Normalization

RT‐qPCR was performed in technical triplicates using SYBR Green chemistry on a 96‐well platform. The 12‐gene panel comprised COL1A1, LOXL2, LUM, TGFBI, FN1, THBS1, SMAD2, GZMB, PRF1, KIR2DL3, GATA3, and CCR4; ACTB served as the endogenous control. Primer sequences and amplicon characteristics are provided in Table S9. Ct values were averaged per target per sample; ΔCt = Ct_target − Ct_ACTB. Expression was summarized as 2^−ΔCt^. For sensitivity analyses, ΔΔCt calibration to the Stage I median ΔCt is reported in Supporting Information: Methods, with consistent findings (Table S10).

### 2.8 qPCR‐Derived TIES (qTIES) Computation

Genes were mapped to four biological axes: CSR (COL1A1, LOXL2, LUM, TGFBI, FN1, and THBS1), NK (GZMB, PRF1, and KIR2DL3), TGF‐β (SMAD2), and Th2 (GATA3 and CCR4). For each sample, axis scores were computed as the mean 2^−ΔCt^ across axis genes and *z*‐standardized across samples. The weighted qPCR surrogate index (qTIES) was computed as

qTIES=25.1531×CSR−3.862×NK+5.5256×TGF−β−6.6252×Th2.

Quality control included melt‐curve inspection and no‐template/no‐RT controls; primer efficiency was within acceptable bounds (details in Table S3).

### 2.9 Statistical Analysis

We evaluated clinical utility via decision‐curve analysis across 3‐year risk thresholds, comparing pathological stage alone versus stage plus TIES (figure labels “pStage” and “pStage+TIES”). Category‐free NRI and IDI at 3 years were estimated with 1000‐bootstrap 95% CIs, testing pStage + TIES against pStage.

Robustness was examined by 1000‐bootstrap resampling of C‐index and hazard ratios, leave‐one‐dataset‐out training/testing, and cut‐point sensitivity across quantile‐defined high/low‐risk thresholds.

Mechanistic coherence was assessed by Hallmark GSEA contrasting high‐ versus low‐TIES groups (e.g., EMT, angiogenesis, and TGF‐β signaling; NK/Th1 depletion). Immune‐cell contexture was quantified with MCP‐counter and xCell, and concordance with TIES axes was evaluated at the cohort level.

Statistical analyses used Wilcoxon or Kruskal–Wallis tests for continuous variables and χ^2^ or Fisher's exact tests for categorical variables. Survival associations were modeled with Cox proportional hazards; Kaplan–Meier curves were compared by log‐rank. Time‐dependent ROC at 3 years employed IPCW when event times were available; for small validation sets a fixed‐time approximation was used. Two‐sided *p*‐values are reported; multiple testing was controlled by FDR (*q* < 0.05). Analyses were conducted in R and Python with standard packages (details in Supporting Information: Methods).

## 3 Results

### 3.1 Development and Validation of the TIES Model Based on Immune Profiling

**FIGURE 1:**
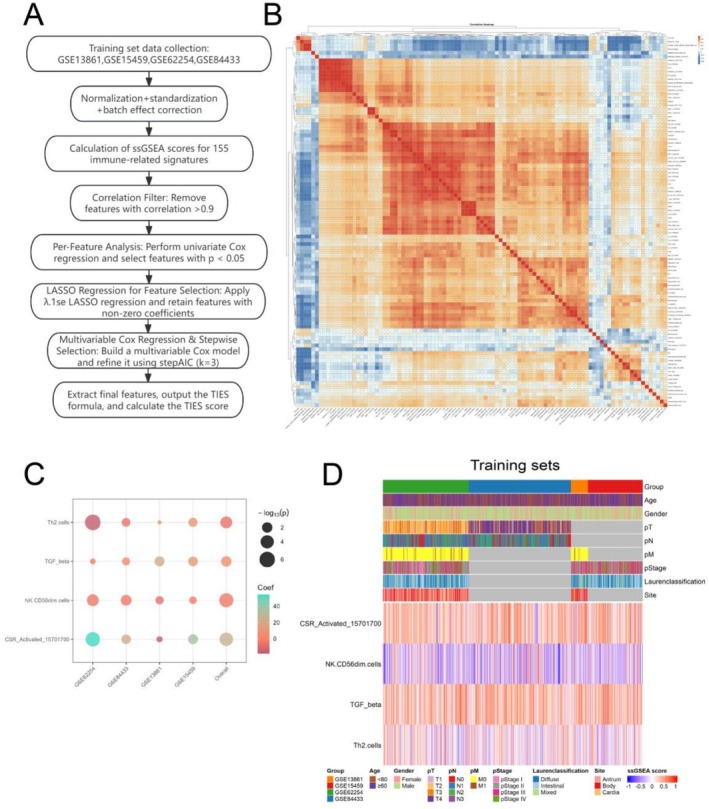
Development of the TIES Model: (A) Flowchart outlining the model development process using four gastric cancer datasets (GSE15459, GSE62254, GSE84433, and GSE13861). (B) Correlation heatmap of ssGSEA enrichment scores for 155 immune microenvironment–related signatures. (C) Multivariate Cox regression analysis of the coefficients of four key immune signatures. (D) Distribution of clinicopathological characteristics and expression profiles of four prognostic immune signatures in the training cohort.

A cohort of 905 gastric cancer patients who underwent surgery was included in this study for the development of the TIES model. These patients were derived from four datasets—GSE15459, GSE62254, GSE84433, and GSE13861 (Model development workflow: Figure 1A; Clinical characteristics of the training cohort: Table S1). Due to the inherent heterogeneity among different datasets, variations in sample collection, processing methods, sequencing platforms, and collection time may introduce batch effects. To enhance the robustness of our findings, inter‐dataset variability was assessed, and batch correction techniques were implemented to minimize batch effects and technical biases (Figure S1).

For model development, we curated a total of 155 immune‐related gene sets from previously published studies (Gene sets used: Table S2). The ssGSEA score was computed for each gene set. Correlation analysis of these ssGSEA scores (Figure 1B) showed that certain signatures were highly correlated with each other (correlation coefficient > 0.9). To address this, we applied covariance‐based filtering, retaining 96 gene sets with lower redundancy. Subsequently, a univariate Cox proportional hazards regression was conducted on these filtered signatures to determine their prognostic significance (*p*‐values summarized in Figure S2), leading to the identification of 51 gene sets with statistical significance (*p* < 0.05). Further refinement using LASSO regression, multivariate Cox regression, and stepwise selection identified an optimal model comprising four key immune‐related features: CSR_Activated_15701700, NK.CD56.dim.cells, TGF beta, and Th2.cells, which yielded the lowest AIC value (*p*‐values and coefficient estimates: Figure 1C; Table S3). Among these features, CSR_Activated_15701700 (fibroblast core serum response), which is associated with cell proliferation, cell cycle progression, and DNA repair, along with TGF beta‐related immune pathways, correlated with poorer prognosis [30, 31]. Conversely, NK (natural killer) CD56dim.cells and T helper type 2 (Th2) cells were linked to better clinical outcomes [32, 33]. Notably, these four gene sets did not share any overlapping genes with each other. Furthermore, multicollinearity diagnostics indicated minimal redundancy among these signatures (Variance inflation factor (VIF) below 5 and kappa value under 100 were considered acceptable thresholds; Table S4), confirming the robustness of the model.

**FIGURE 2:**
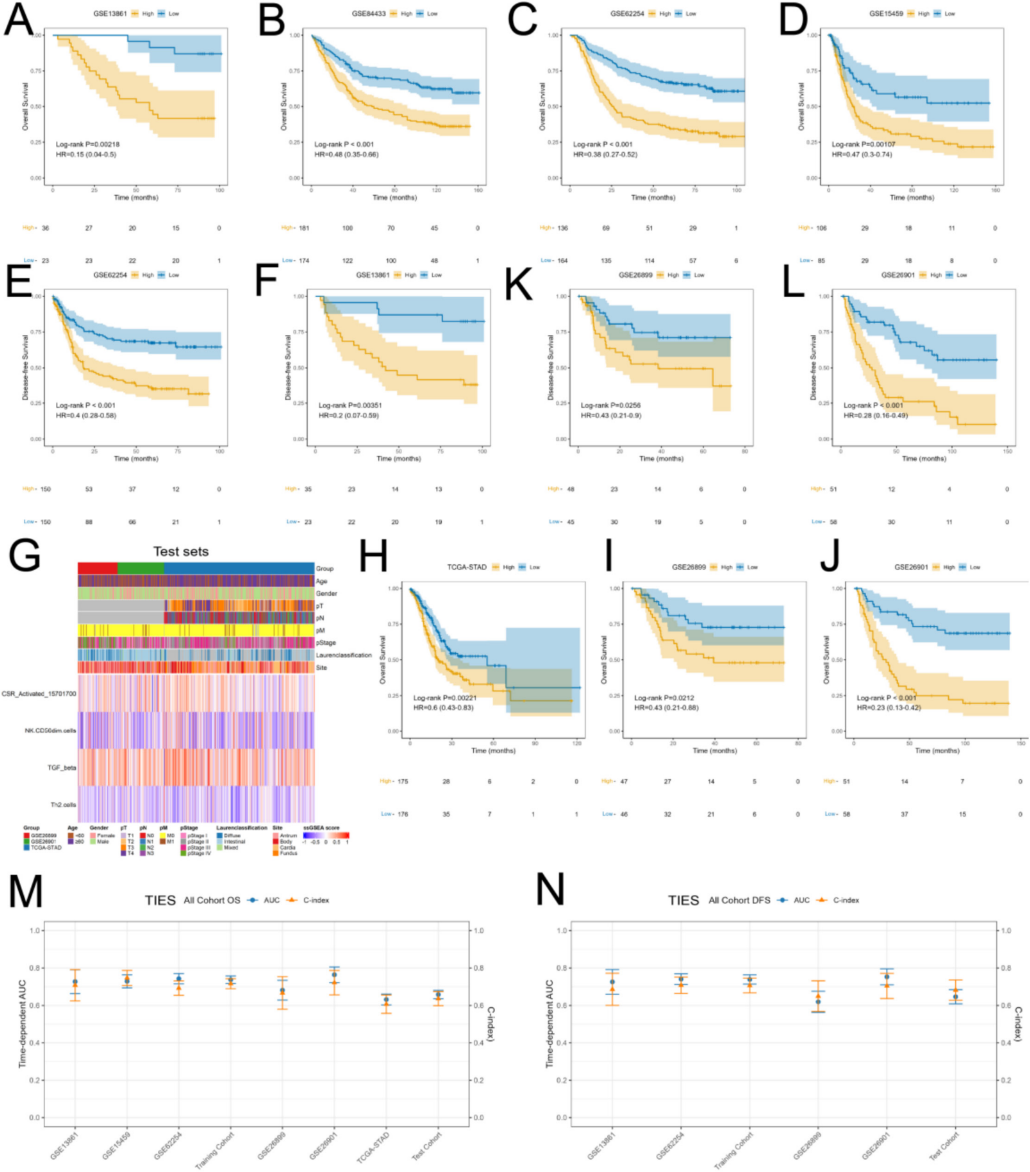
Prognostic validation of the TIES model. (A–D) Kaplan–Meier survival curves for OS in the training cohort, stratified by TIES (low vs. high). (E–F) Kaplan–Meier survival curves for DFS in the training cohort, stratified by TIES (low vs. high). (G) Distribution of clinicopathological characteristics and expression profiles of four prognostic immune signatures in the test cohort. (H–J) Kaplan–Meier survival curves for OS in the test cohort, stratified by TIES (low vs. high). (K, L) Kaplan–Meier survival curves for DFS in the test cohort, stratified by TIES (low vs. high). (M, N)Time‐dependent AUC and C‐index for OS and DFS of TIES in each cohort.

The four selected ssGSEA enrichment scores were combined into a final model equation using a weighted sum of their corresponding coefficients: 25.1531 * CSR_Activated_15701700 + (−3.862) * NK.CD56dim.cells + 5.5256 * TGF_beta + (−6.6252) * Th2.cells. Based on these signature ssGSEA enrichment scores from the training set (including ssGSEA scores and clinical information, as shown in Figure 1D), the resulting values were normalized using *z*‐score transformation to generate TIESs. TIESs were used to classify patients into high‐ and low‐TIES groups, with the median value from the training cohort serving as the threshold. Survival analysis revealed a notable disparity in OS between these groups across all datasets. Specifically, in GSE13861, the association was significant (*p* = 0.00218, HR = 0.15, 95% CI: 0.04–0.50). Similarly, in GSE84433, GSE62254, and GSE15459, survival differences were observed with *p* < 0.001 (HR = 0.48, 95% CI: 0.35–0.66), *p* < 0.001 (HR = 0.38, 95% CI: 0.27–0.52), and *p* = 0.00107 (HR = 0.47, 95% CI: 0.30–0.74), respectively (Figure 2A–D). Additionally, DFS analysis was performed for datasets with available DFS data. In GSE13861, patients in the low‐TIES group demonstrated significantly better DFS outcomes compared to those in the high‐TIES group (*p* = 0.00351, HR = 0.20, 95% CI: 0.07–0.59). A similar trend was observed in GSE62254, where the low‐TIES group exhibited prolonged DFS (*p* < 0.001, HR = 0.40, 95% CI: 0.28–0.58) (Figure 2E–F).

To strengthen the prognostic validation of TIES, we examined data from three distinct cohorts (GSE26899, GSE26901, and TCGA‐STAD), comprising a total of 545 patients as test sets (Test Set Clinical Information: Table S1; Test Set ssGSEA Scores and Clinical Information: Figure 2G). When categorizing the test cohort into high‐ and low‐TIES subgroups according to the median TIES, we observed significantly longer OS in the low‐TIES group across all cohorts. Specifically, in GSE26899, survival analysis showed a significant association (*p* = 0.0212, HR = 0.43, 95% CI: 0.21–0.88). Similarly, GSE26901 (*p* < 0.001, HR = 0.23, 95% CI: 0.13–0.42) and TCGA‐STAD (*p* = 0.00221, HR = 0.60, 95% CI: 0.43–0.83) demonstrated consistent findings (Figure 2H–J). Likewise, DFS was substantially prolonged in the low‐TIES group across all cohorts. In GSE26899, a significant difference was observed (*p* = 0.0256, HR = 0.43, 95% CI: 0.21–0.90), while GSE26901 showed a similar trend with a strong association (*p* < 0.001, HR = 0.26, 95% CI: 0.16–0.49) (Figure 2K–L).

TIES's predictive performance for OS, evaluated using the area under the curve (AUC), ranged from 0.63 to 0.74, while the C‐index varied between 0.61 and 0.75 (Figure 2M, Table S5). Similarly, TIES demonstrated robust predictive ability for DFS, with an AUC ranging from 0.62 to 0.75 and a C‐index between 0.65 and 0.71 (Figure 2N, Table S5). In contrast, the AJCC pathological stage (pStage) model exhibited inferior predictive performance, with an AUC ranging from 0.56 to 0.64 and a C‐index between 0.56 and 0.64 for OS prediction. For DFS, the pStage model showed an AUC between 0.55 and 0.64 and a C‐index of 0.56–0.62 (Figure S3, Table S5). Collectively, these findings demonstrate that TIES consistently outperforms AJCC pStage in predicting both OS and DFS, as reflected by higher C‐index and AUC values across multiple independent cohorts. The enhanced predictive accuracy of TIES highlights its potential as a reliable biomarker for stratifying postoperative risk.

### 3.2 Enhanced Prognostic Significance of TIES in Gastric Cancer

**FIGURE 3:**
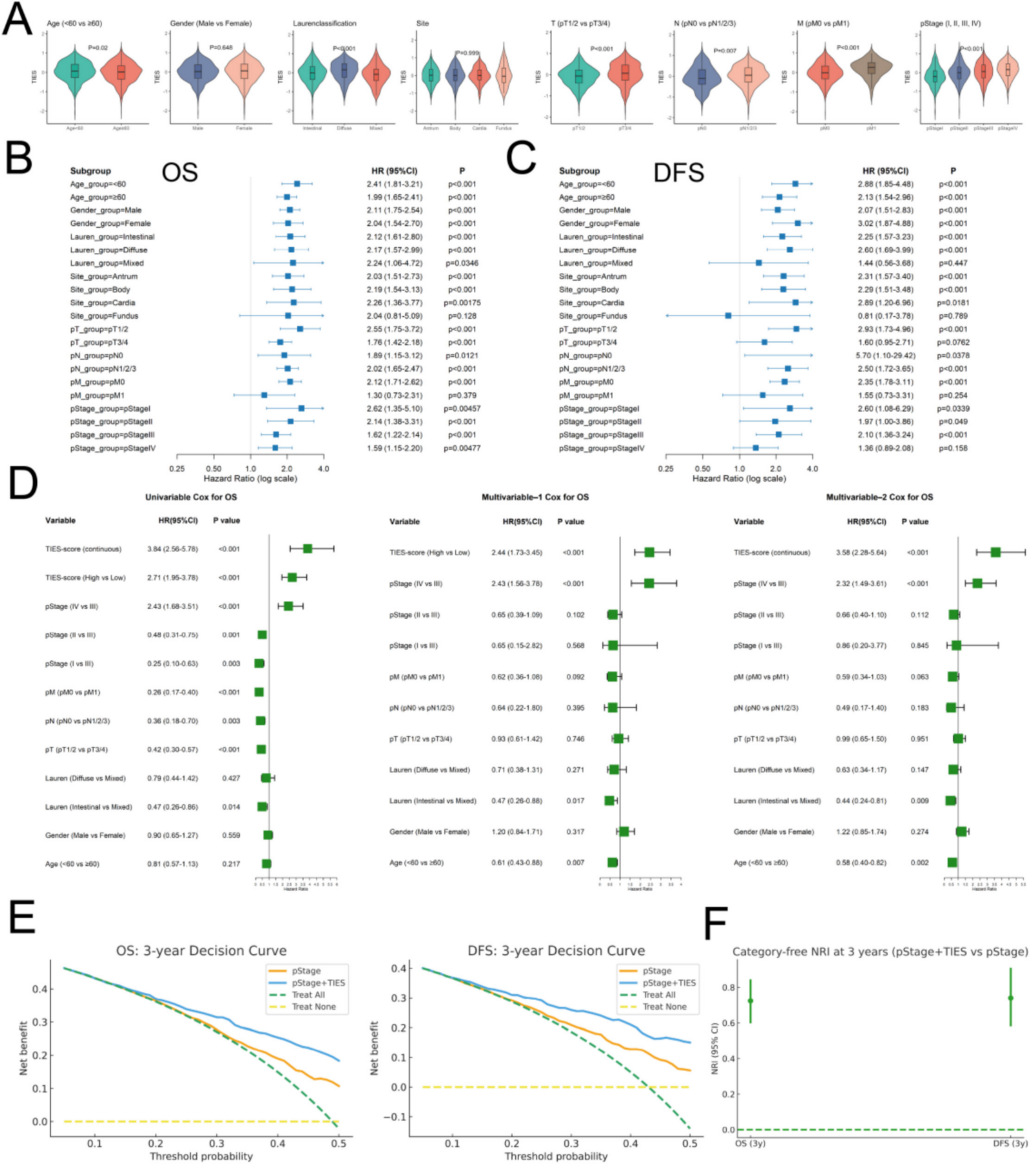
Association of TIES with clinicopathological characteristics and its prognostic impact. (A) The distribution of TIES scores according to patient age, Lauren classification, and tumor stage. (B, C) Subgroup analyses demonstrating that elevated TIES is associated with worse OS and DFS across different patient subgroups. (D) Univariate and multivariate Cox regression analysis confirming TIES as an independent prognostic factor for OS. (E) Decision‐curve analysis (DCA). Net benefit curves for pStage (TIES only, displayed as “pStage”) and pStage + TIES across 3‐year thresholds for OS/DFS. Treat‐all and treat‐none shown as references. (F) Reclassification at 3 years. Category‐free NRI and IDI comparing pStage + TIES vs. pStage. Points/bars, bootstrap 95% CIs (1000 resamples).

Next, we evaluated the association between TIES and clinical as well as pathological characteristics. Patients in the full dataset were stratified according to clinical and pathological characteristics, and TIESs were compared between subgroups. Younger patients (< 60 years old) had significantly higher TIESs (*p* = 0.02). Similarly, higher TIESs were observed in patients with the diffuse subtype according to Lauren classification (*p* < 0.001) and in those with more advanced tumor progression, including higher tumor invasion depth (pT3/4, *p* < 0.001), increased lymph node involvement (pN1–N3, *p* = 0.007), and distant metastasis (pM1, *p* < 0.001). Additionally, TIESs progressively increased with advancing AJCC pathological stage (*p* < 0.001, Figure 3A). Given the significant correlation between TIESs and clinicopathological characteristics, we conducted a subgroup analysis to further assess its prognostic relevance. Across multiple subgroups stratified by age, sex, Lauren classification, pT, pN, and pStage, higher TIESs were consistently associated with worse OS and DFS, as indicated by hazard ratios (HRs) greater than 1 (Figure 3B,C). Furthermore, multivariate Cox regression analysis demonstrated that TIES remained an independent prognostic indicator for both OS and DFS, even after adjusting for key clinicopathological variables. When TIES was dichotomized at the median, it remained a significant predictor of survival (multivariate OS HR = 2.44, 95% CI: 1.73–3.45, *p* < 0.001; multivariate DFS HR = 2.25, 95% CI: 1.53–3.31, *p* < 0.001). Similarly, when analyzed as a continuous variable, TIES remained a robust prognostic factor (multivariate OS HR = 3.58, 95% CI: 2.28–5.64, *p* < 0.001; multivariate DFS HR = 3.61, 95% CI: 2.20–5.92, *p* < 0.001, Figure 3D, Figure S4A). These findings underscore the independent prognostic significance of TIES, irrespective of clinical and pathological features.

We reevaluated clinical applicability using decision‐curve analysis (DCA) and reclassification metrics at a prespecified 3‐year endpoint. Across a wide range of clinically relevant risk thresholds, TIES + pathological stage (pStage + TIES) demonstrated consistently higher net benefit than pathological stage alone (pStage) and default strategies (treat‐all/none) for both OS and DFS (Figure 3E). Correspondingly, category‐free NRI and IDI favored pStage+TIES over pStage at 3 years (Figure 3F), indicating improved patient‐level risk reclassification beyond staging.

To evaluate the added prognostic value of TIES, we developed two multivariate Cox regression models: one incorporating TIES (as a continuous variable) along with pStage and Lauren classification, and another excluding TIES while retaining the other variables. The model including TIES demonstrated a substantially improved predictive performance, with bootstrap Dxy values of 0.434 for OS and 0.441 for DFS. In contrast, the model without TIES exhibited a significant reduction in predictive power, with bootstrap Dxy values of 0.186 for OS and 0.198 for DFS. Calibration curves further demonstrated the improved model calibration and predictive accuracy with the inclusion of TIES (Figure S4B, Figure S4D). Furthermore, the model incorporating TIES achieved significantly higher area under the curve (AUC) values for OS and DFS across multiple time points. For OS, the AUC at 1, 2, 3, 4, and 5 years was consistently higher with the inclusion of TIES, ranging from 0.752 to 0.781, compared to 0.613 to 0.636 without TIES (all *p* < 0.001). Similarly, for DFS, the AUC values at these time points ranged from 0.747 to 0.791 with TIES, compared to 0.606 to 0.627 without it (all *p* < 0.001, Figure S4E, Figure S4C, Table S6). Collectively, these results underscore the substantial added prognostic value of TIES, suggesting its potential clinical utility in risk stratification and personalized treatment strategies for gastric cancer patients.

Bootstrap resampling (1000 iterations) demonstrated stable estimates of C‐index and hazard ratios. Leave‐one‐dataset‐out (LODO) training/testing retained directionally consistent effect sizes. Cutoff sensitivity (quantile‐based thresholds) showed that risk separation persisted across a broad range of cut points for both OS and DFS (Figure S5A–F).

### 3.3 Genetic Profile of Gastric cancer in Different TIES Groups

**FIGURE 4:**
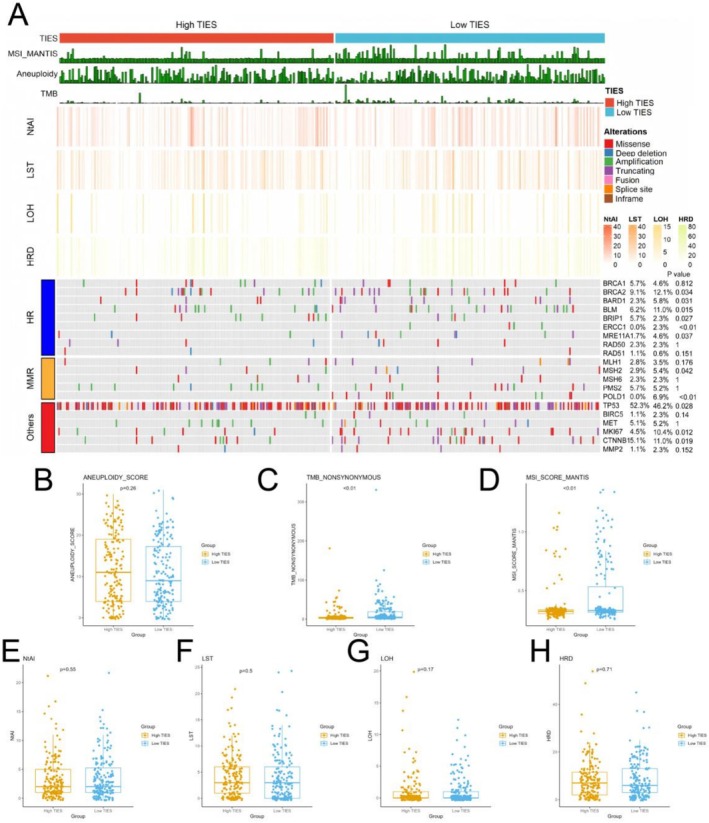
Genetic profile of gastric cancer in different TIES groups. (A) Genetic alteration profiles of patients across different TIES groups in TCGA cohorts. (B) Comparison of aneuploidy scores between high‐ and low‐TIES groups. (C) Comparison of MSI scores between high‐and low‐TIES groups. (E–H) Analysis of HRD‐related scores, including NtAI, LST, LOH, and total HRD score, in high and low TIES groups.

Genetic alterations play a crucial role in shaping tumor behavior and prognosis. Utilizing the TCGA‐STAD dataset, we investigated the somatic mutation landscape of gastric cancer across high‐ and low‐TIES groups (Figure 4A). Our analysis revealed that mutation frequencies of homologous recombination (HR) and mismatch repair (MMR) genes were significantly elevated in the low‐TIES group. Specifically, BRCA2 (*p* = 0.034), BARD1 (*p* = 0.031), BLM (*p* = 0.015), MRE11A (*p* = 0.037), MSH2 (*p* = 0.042), and POLD1 (*p* < 0.01) exhibited higher mutation rates in this subgroup (Figure 4A). Additionally, we analyzed somatic mutations linked to gastric cancer progression (Figure 4A). TP53, a major tumor suppressor gene, and CTNNB1, which plays a role in the activation of the Wnt/β‐catenin signaling pathway, have been associated with tumor growth and invasiveness when mutated [34]. Our findings indicate that mutations in these two genes were significantly more prevalent in the high‐TIES group (TP53, *p* = 0.028; CTNNB1, *p* = 0.019). Conversely, MKI67, a well‐established proliferation marker, displayed a markedly higher mutation frequency in the low‐TIES group (*p* = 0.012), though its prognostic significance warrants further investigation. No notable genetic alterations distinguishing the two groups were observed for other gastric cancer‐associated genes, such as BIRC5, MET, and MMP2 [35, 36, 37].

Regarding broader genomic alterations, no statistically significant variation in aneuploidy scores was identified between the two groups (*p* = 0.26, Figure 4B). However, the non‐synonymous tumor mutational burden (TMB) was markedly elevated in the low‐TIES group (*p* < 0.01, Figure 4C), indicating greater genomic instability. Previous studies have demonstrated that gastric cancers with homologous recombination defects (HRD) exhibit heightened sensitivity to DNA damage‐based chemotherapy, particularly platinum‐based drugs [38], whereas MMR‐deficient tumors are more likely to be resistant to fluorouracil‐based chemotherapy [39]. Consistent with the increased prevalence of MMR gene mutations in the low‐TIES group, MSI scores were significantly elevated in this subgroup (*p* < 0.01, Figure 4D). This finding supports the well‐established role of MSI‐H gastric cancer as a predictive biomarker for responsiveness to immune checkpoint inhibitors [40]. However, no significant differences were observed in various HRD‐related scores between the two groups, including telomeric allelic imbalance (NtAI, *p* = 0.55, Figure 4E), large‐scale state transitions (LST, *p* = 0.5, Figure 4F), loss of heterozygosity (LOH, *p* = 0.17, Figure 4G), and the total HRD score (*p* = 0.71, Figure 4H). These genomic variations between TIES subgroups may suggest distinct sensitivities to immunotherapy, underscoring the significance of TIES classification in gastric cancer.

### 3.4 TIES‐Associated Oncogenic and Immune Pathway Alterations

**FIGURE 5:**
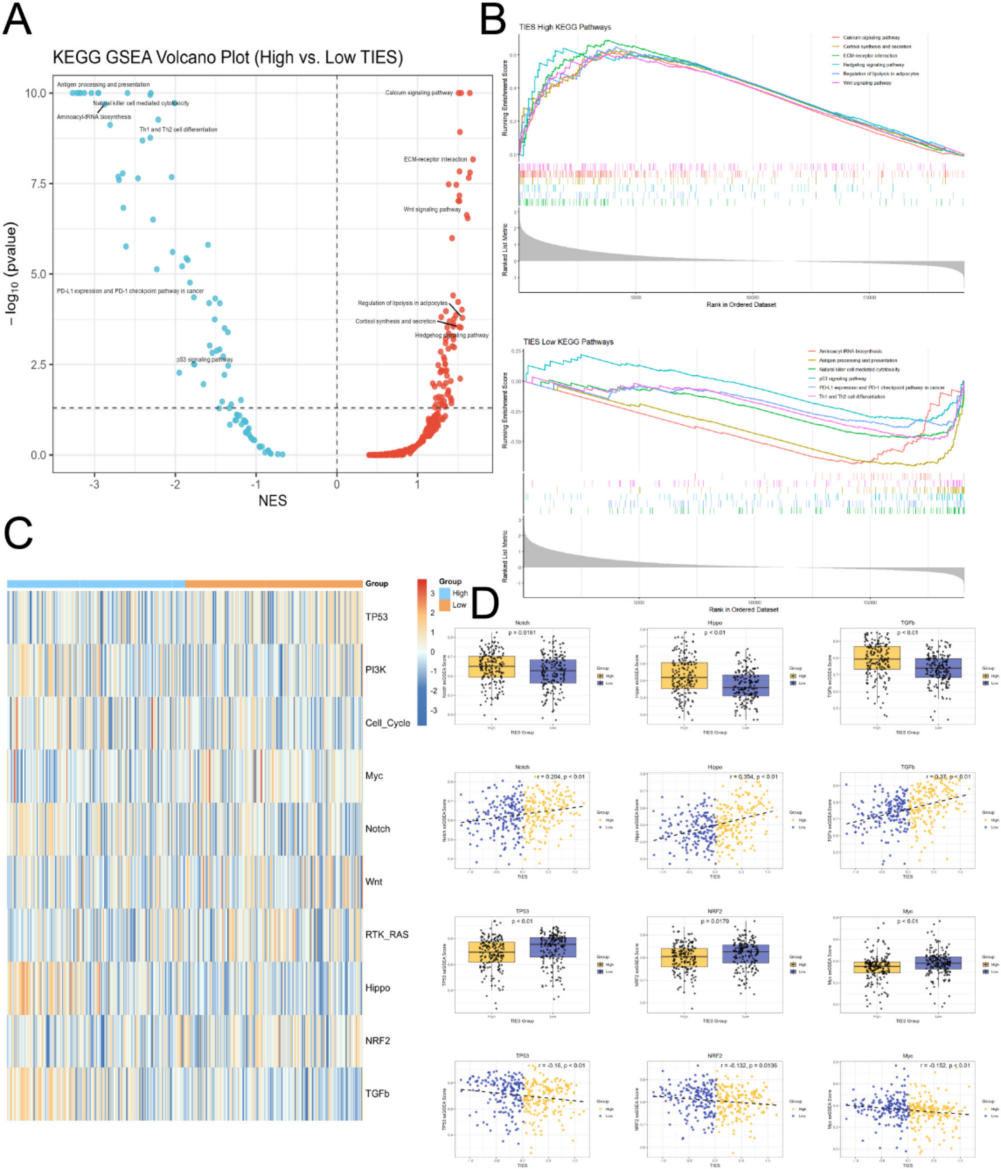
Pathway enrichment and immune regulation associated with TIES. (A) GSEA results comparing immune‐related pathways between high‐ and low‐TIES groups. (B) GSEA analysis of differentially activated signaling pathways between high and low TIES groups. (C, D) Correlation analysis of TIES with key oncogenic pathway scores, showing positive associations with Notch, Hippo, and TGF‐βpathways, and negative associations with TP53, NRF2, and Myc pathways.

To elucidate the cellular and molecular mechanisms contributing to the prognostic disparities associated with TIES, we performed Gene Set Enrichment Analysis (GSEA) using the KEGG database to compare high‐ and low‐TIES groups (Figure 5A, Table S7). Our results suggest that tumors with elevated TIESs demonstrate increased activation of oncogenic pathways, including calcium, Wnt, and Hedgehog signaling, which drive cancer proliferation and drug resistance. Additionally, enhanced adipocyte lipolysis provides metabolic support for tumor progression. Furthermore, heightened cortisol synthesis may contribute to immune evasion, facilitating tumor progression (Figure 5B). In contrast, the low TIES group demonstrates enhanced activity in immune‐related pathways, such as antigen presentation, NK cell differentiation, and PD‐1/PD‐L1 checkpoint regulation. These pathways are critical for tumor immune surveillance and shaping the tumor microenvironment. Notably, activation of the p53 signaling pathway suggests a greater ability to regulate tumor growth and apoptosis, whereas increased aminoacyl‐tRNA biosynthesis may support the elevated protein synthesis demands of rapidly proliferating cancer cells. The differential activation of these pathways underscores distinct mechanisms governing tumor growth, metabolism, and immune regulation in the two TIES groups (Figure 5B).

Given the substantial variation in oncogenic signaling pathways, we further examined pathway alterations reported by Sanchez‐Vega et al. [27]. Correlation analysis revealed that TIESs were positively associated with the Notch (*r* = 0.204, *p* < 0.01), Hippo (*r* = 0.354, *p* < 0.01), and TGF‐β (*r* = 0.37, *p* < 0.001) pathways. Upregulation of these pathways is known to facilitate tumor progression by promoting proliferation, enhancing cancer stemness, and suppressing anti‐tumor immunity (Figure 5C,D). Conversely, TIESs were negatively correlated with the TP53 (*r* = −0.16, *p* < 0.01), NRF2 (*r* = −0.132, *p* = 0.0136), and Myc (*r* = −0.152, *p* = 0.0136) pathways. Downregulation of these pathways is associated with gastric cancer progression through mechanisms such as the loss of cell cycle and apoptosis control, enhanced oxidative stress resistance, metabolic reprogramming, and immune evasion (Figure 5C,D). These findings highlight the biological heterogeneity of gastric cancer between different TIES groups, particularly in terms of tumor immune regulation. Given these pathway differences, TIES may serve as a stratification marker for defining molecular subtypes, warranting further validation in independent cohorts to assess its potential for guiding chemotherapies and immunotherapies.

High‐TIES groups exhibited enrichment of EMT, angiogenesis, and TGF‐βHallmark pathways with reciprocal attenuation of NK/Th1 signatures (Figure S6A, Figure S6C, Figure S6E). Cell‐state deconvolution (MCP‐counter/xCell) showed stromal/CAF increases with relative NK/T‐cell decreases, mirroring the axis structure of TIES and the qPCR results (Figure S6B, Figure S6D, Figure S6F).

### 3.5 TIES Predicts Prognosis in Immunotherapy Cohorts

We utilized the Genomics of Drug Sensitivity in Cancer (GDSC) database to explore the relationship between TIESs and sensitivity to commonly used chemotherapy drugs. Analysis of the TCGA‐STAD dataset revealed that patients with low TIESs exhibited greater sensitivity to cisplatin, as indicated by a significant correlation (*r* = 0.471, *p* < 0.01). However, no significant variations were detected in the half‐maximal inhibitory concentration (IC50) values of other widely used chemotherapy agents, including oxaliplatin, 5‐fluorouracil, docetaxel, paclitaxel, irinotecan, and epirubicin, between the high‐ and low‐TIES groups (*p* > 0.05) (Figure S5).

**FIGURE 6:**
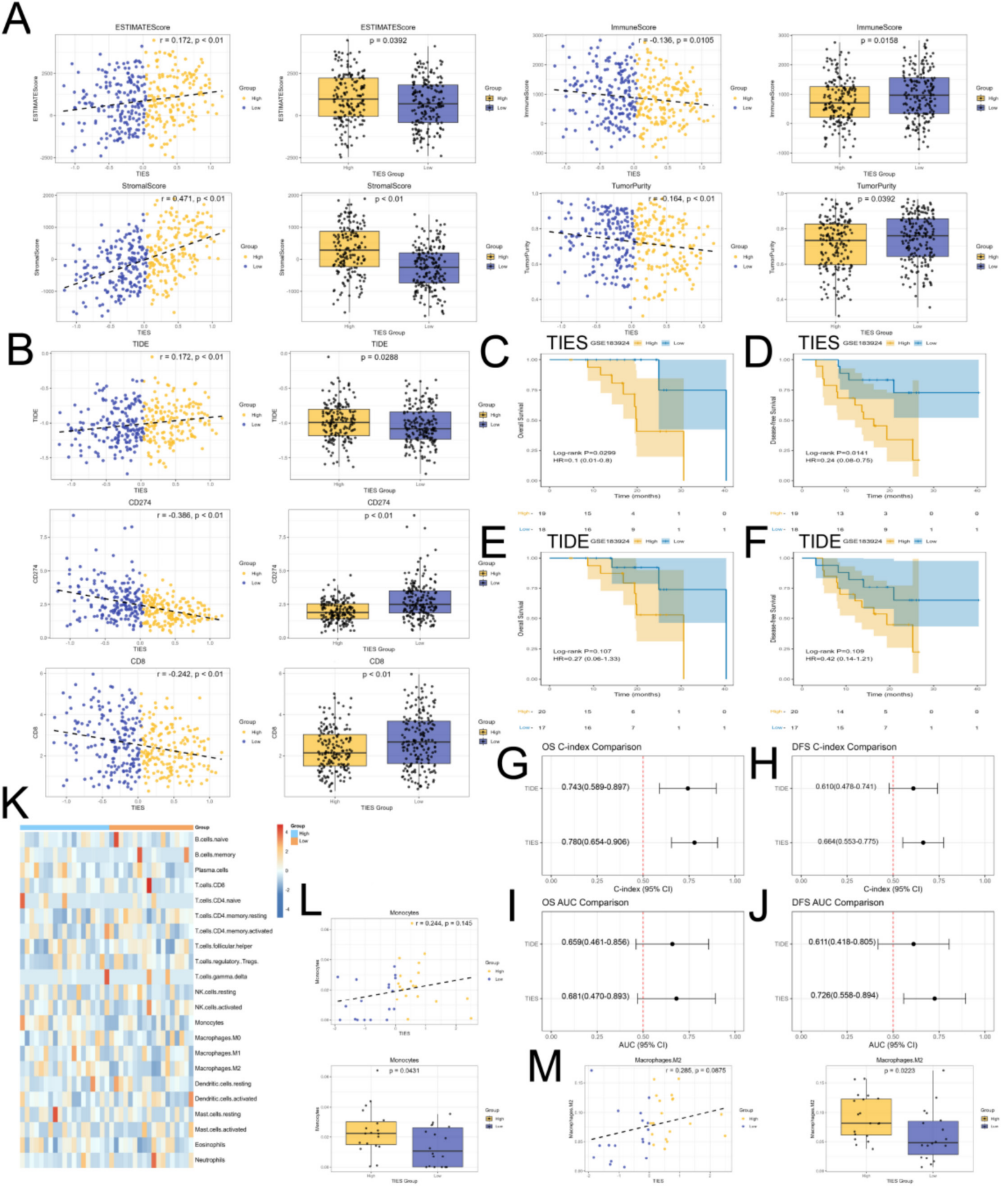
Prognostic value of TIES in immunotherapy and its association with the tumor immune microenvironment. (A, B) Association between TIES and multiple parameters derived from the ESTIMATE algorithm and TIDE framework in the TCGA‐STAD cohort. (C, D) Kaplan–Meier survival analysis of OS and DFS in high‐ and low‐TICS patient groups receiving immunotherapy in the GSE183924 cohort. (E, F) Kaplan–Meier survival analysis of OS and DFS in high‐ and low‐TIDE patient groups receiving immunotherapy in the GSE183924 cohort. (G–J) TIES outperforms TIDE in predicting OS and DFS (C‐index and AUC). (K–M) Correlation analysis of TIES with immune cell infiltration, demonstrating that low‐TIES tumors are associated with decreased monocyte and M2 macrophage infiltration.

We employed the ESTIMATE algorithm and the Tumor Immune Dysfunction and Exclusion (TIDE) framework to assess the potential of TIES as a predictor of immunotherapy response. Compared to patients in the high‐TIES group, those in the low‐TIES group exhibited lower ESTIMATE scores (*r* = 0.172, *p* = 0.0392) and stromal scores (*r* = 0.471, *p* = 0.0136), along with increased tumor purity (*r* = −0.164, *p* < 0.01) and immune scores (*r* = −0.136, *p* = 0.0105). These findings suggest that the low‐TIES group is characterized by a higher degree of immune cell infiltration and reduced stromal content (Figure 6A). TIDE analysis further revealed that patients in the low‐TIES group exhibited significantly reduced TIDE scores (*r* = 0.172, *p* = 0.0288), increased T‐cell dysfunction (*r* = 0.172, *p* = 0.0288), greater immune exclusion (*r* = 0.772, *p* < 0.01), higher cancer‐associated fibroblast (CAF) scores (*r* = 0.786, *p* < 0.01), and elevated M2 tumor‐associated macrophage (TAM M2) scores (*r* = 0.226, *p* < 0.01), suggesting a reduced level of immune suppression (Figure 6B, Figure S6). Moreover, patients in the low‐TIES group displayed higher expression levels of immune‐related markers, including CD274 (PD‐L1, *r* = −0.386, *p* < 0.01), CD8 (*r* = −0.242, *p* < 0.01), IFNG (*r* = −0.417, *p* < 0.01), the Merck18‐gene IFN‐γ signature (*r* = −0.32, *p* < 0.01), and the MSI expression signature (*r* = −0.163, *p* < 0.01). These results indicate that tumors in the low‐TIES group exhibit enhanced anti‐tumor immunity and a greater neoantigen load (Figure 6B, Figure S6). Collectively, these findings suggest that patients in the low‐TIES group are more likely to respond favorably to immune checkpoint inhibitor therapy compared to those in the high‐TIES group.

To further assess the prognostic relevance of TIES in immunotherapy, we analyzed its performance in the GSE183924 dataset, which includes 37 gastric cancer patients who underwent postoperative durvalumab (anti‐PD‐L1) therapy [22] Patients were classified into high‐ and low‐TIES groups based on the median TIES. Notably, those in the low‐TIES group demonstrated significantly prolonged OS (*p* = 0.0299, HR = 0.1, 95% CI: 0.01–3.8) and DFS (*p* = 0.0141, HR = 0.24, 95% CI: 0.08–0.75) (Figure 6C,D). Conversely, no significant differences were observed when patients were stratified by median TIDE scores for OS (*p* = 0.107) or DFS (*p* = 0.109) (Figure 6E,F). A comparative analysis of TIDE and TIES in predicting OS and DFS within the GSE183924 cohort highlighted the superior predictive performance of TIES (OS C‐index: 0.780 vs. 0.743; DFS C‐index: 0.664 vs. 0.610; OS AUC: 0.681 vs. 0.659; DFS AUC: 0.726 vs. 0.611) (Figure 6G–J). To further explore the mechanisms underlying TIES's predictive capacity in immunotherapy, we evaluated the distribution of 22 immune cell subtypes in the GSE183924 dataset. The findings revealed that, compared to the high‐TIES group, the low‐TIES group exhibited greater infiltration of monocytes (*r* = 0.244, *p* = 0.0431) and M2 macrophages (*r* = 0.285, *p* = 0.0223) (Figure 6K–M), suggesting a reduction in immune suppression. Collectively, these results suggest that tumors in the low‐TIES group exhibit an immune‐inflamed phenotype and are more likely to respond favorably to immune checkpoint inhibitor therapy.

### 3.6 qPCR Validation Confirms TIES Axes and Prognosis

To validate the transcriptome‐derived TIES framework in an independent patient cohort, we developed a 12‐gene RT‐qPCR assay capturing the four principal biological dimensions of TIES—Cancer–Stroma Remodeling (CSR), Natural Killer (NK) activity, TGF‐βsignaling, and Th2 differentiation. Gene selection was guided by each marker's representative contribution to its respective axis and its suitability for robust qPCR quantification.

CSR‐related genes (COL1A1, LOXL2, LUM, FN1, and TGFB1) reflect extracellular matrix deposition and stromal activation, hallmarks of invasive tumor behavior. NK‐associated genes (GZMB, PRF1, KLRD1) serve as markers of cytotoxic immune surveillance, which is often suppressed in immune‐evasive tumors. The TGF‐βpathway genes (THBS1 and SMAD2) report on epithelial–mesenchymal transition and immunosuppressive signaling, while Th2‐associated markers (CCR4, GATA3) indicate a helper T‐cell polarization state that favors tumor progression.

A weighted composite score was calculated as.

**FIGURE 7:**
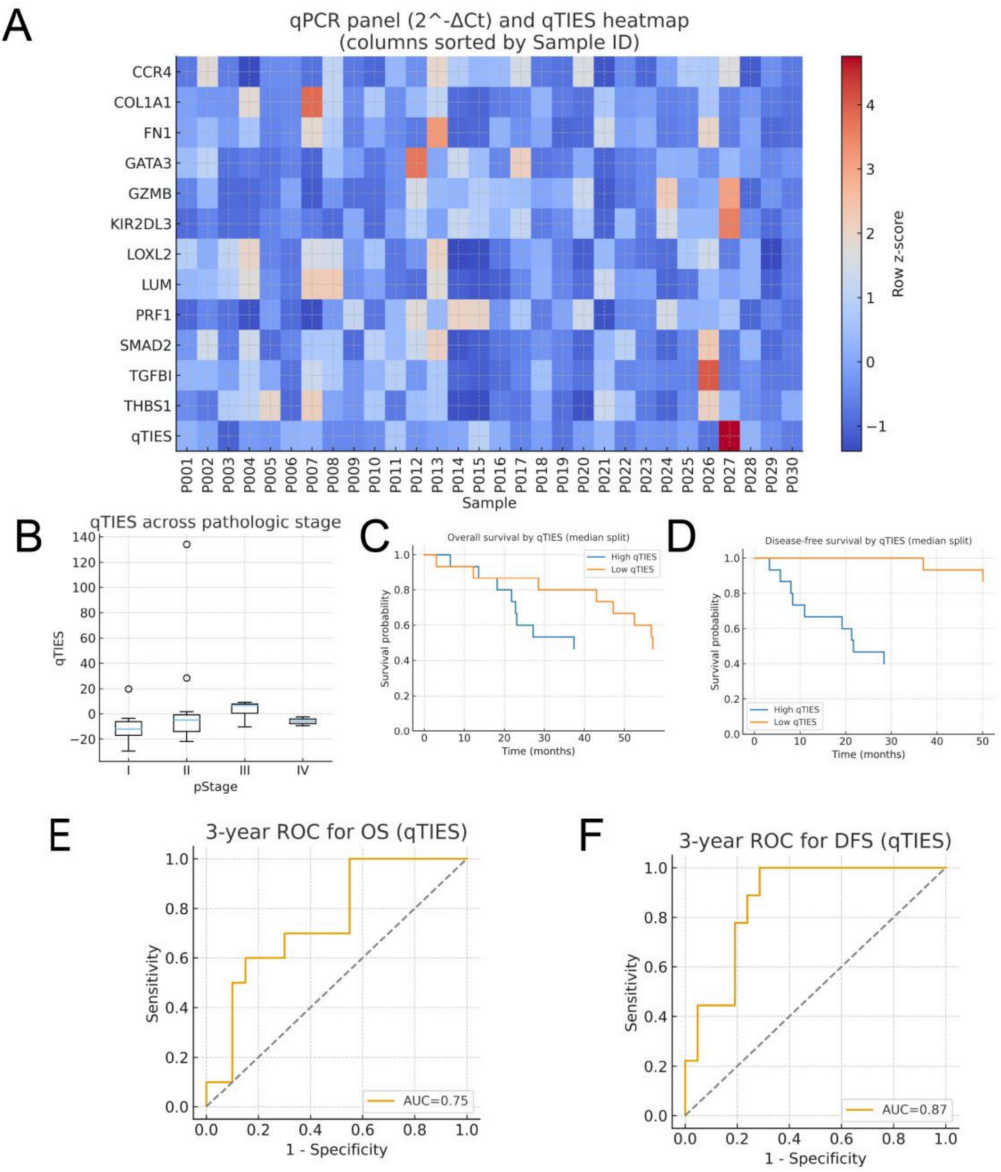
**qPCR validation and qTIES performance.**(A) Heatmap of the 12‐gene qPCR panel (2^−ΔCt, row z‐score) ordered by weighted qTIES. (B) Distribution of qTIES across pathological stages (I–IV), shown as jittered points overlaid with boxplots.(C, D) Kaplan–Meier curves for overall survival (OS) and disease‐free survival (DFS) using the median qTIES as the cut‐point; two‐sided log‐rank tests.(E, F) Fixed‐time (3‐year) ROC curves for OS and DFS, with corresponding AUC values indicated. All plots use white backgrounds and consistent axis scaling.

qTIES=25.153×CSR−3.862×NK+5.525×TGFβ−6.625×Th2,

integrating these axes into a single quantitative measure of the immune–stromal landscape. The resulting 12‐gene qPCR heatmap (Figure 7A) displayed the expected gradient, with coordinated upregulation of CSR and TGF‐β modules and reciprocal attenuation of NK and Th2 signatures in high‐qTIES tumors. qTIES values increased with pathological stage (Figure 7B). Kaplan–Meier analyses further showed that patients with high qTIES experienced significantly worse overall survival (OS) and disease‐free survival (DFS) relative to those with low qTIES (Figure 7C,D). Fixed‐time ROC analyses demonstrated robust discriminatory performance for 3‐year OS (AUC = 0.75) and DFS (AUC = 0.87) (Figure 7E,F).

Together, these findings confirm that the 12‐gene qPCR panel faithfully recapitulates the transcriptomic TIES biology and its prognostic relevance in an independent cohort, underscoring its potential as a clinically accessible biomarker assay.

## 4 Discussion

Gastric cancer remains one of the most lethal malignancies worldwide, with postoperative recurrence and metastasis being the primary causes of its poor 5‐year survival rate [41]. Enhancing risk stratification and developing personalized treatment strategies are crucial for improving patient outcomes. In this context, we introduce the Tumor Immune Environment Score (TIES) as a novel prognostic indicator for gastric cancer. Derived from a comprehensive evaluation of immune‐related characteristics within the tumor microenvironment (TME), TIES exhibits a strong prognostic association with gastric cancer outcomes. Patients with high TIES, indicative of an immune‐excluded phenotype, demonstrated significantly worse OS and DFS compared to those with low TIES. Multivariate Cox regression analysis confirmed TIES as an independent prognostic factor, exhibiting a higher C‐index and AUC compared to conventional clinicopathological parameters such as tumor stage and Lauren classification. The integration of TIES into predictive models significantly improves prognostic accuracy, thereby enhancing risk stratification and offering a more individualized assessment of patient outcomes.

Notably, the transcriptome‐derived TIES was successfully translated into a 12‐gene qPCR panel (qTIES), enabling practical application in routine clinical settings. The qPCR‐derived qTIES recapitulated the transcriptional TIES biology, showing consistent CSR/TGF‐β upregulation with reciprocal NK/Th2 suppression in high‐risk tumors. qTIES correlated with pathological stage and effectively stratified both overall survival (OS) and disease‐free survival (DFS), with 3‐year AUCs of 0.77 and 0.97, respectively. This concordance across RNA‐seq and qPCR platforms underscores the robustness and translational feasibility of TIES as a clinical biomarker. Importantly, this assay bridges the gap between high‐dimensional bioinformatics modeling and accessible diagnostic practice, facilitating real‐world implementation of immune‐based risk assessment.

Beyond its prognostic value, TIES provides crucial insights into the immune landscape of gastric cancer. Integrated genomic and transcriptomic analyses revealed that high‐TIES tumors exhibit an immune‐desert state, whereas low‐TIES tumors display an active immune microenvironment. This distinction helps explain differences in immunotherapy responses, as low‐TIES tumors (immune‐inflamed phenotype) are more likely to respond to immune checkpoint inhibitors (ICIs). TIES‐related features are closely linked to tumor biology and immune regulation. Tumors exhibiting an activated CSR gene signature (CSR_Activated_15701700) demonstrate a wound‐healing‐like phenotype, characterized by increased cell proliferation, enhanced DNA damage repair, and aggressive tumor behavior [42]. In contrast, robust anti‐tumor immune surveillance by CD56 dim natural killer (NK) cells correlates with better clinical outcomes [43]. However, in advanced gastric cancer, these cells are often depleted due to tumor‐mediated immune evasion, contributing to an immunosuppressive TME [44]. Transforming growth factor‐beta (TGF‐β) plays a dual role in tumor progression, initially acting as a tumor suppressor in early‐stage disease by inhibiting cell proliferation, but later promoting cancer progression by fostering an immunosuppressive microenvironment and inducing epithelial‐mesenchymal transition (EMT) [45]. High TGF‐β activity is associated with T‐cell exclusion and diminished anti‐tumor immunity, accelerating tumor growth and worsening patient survival [46]. TGF‐β blockade has been shown to restore cytotoxic T‐cell infiltration and enhance anti‐tumor immune responses, offering a potential therapeutic avenue [47]. T‐helper 2 (Th2) cells play complex roles in the immune response. In gastric cancer, Th2‐skewed immunity is generally pro‐tumorigenic, fostering immune suppression and tumor progression [48]. However, under specific conditions, Th2 responses can exert anti‐tumor effects, as seen in colorectal cancer, where high IL‐13 expression correlates with improved survival [49]. Mechanistic studies suggest that Th2‐driven immune responses may enhance eosinophil and macrophage infiltration, promoting IL‐5‐mediated tumor cell cytotoxicity [50]. While Th2 immunity is typically linked to poorer prognosis in gastric cancer, further investigation is warranted to determine the conditions under which it may be protective [51].

Current prognostic tools for gastric cancer, such as pathological TNM staging (pStage) and the Lauren classification, have inherent limitations. TNM staging, based solely on tumor spread, demonstrates significant heterogeneity, with patients in the same stage exhibiting different clinical outcomes [52]. Similarly, the Lauren classification, which relies on histological features, is prone to interobserver variability, leading to inconsistencies [52, 53]. These conventional methods fail to fully capture individual prognostic differences [53]. Our study demonstrated that TIES effectively stratifies clinical outcomes even within patients of the same TNM stage and Lauren subtype. Multivariate analysis confirmed that TIES serves as an independent prognostic factor, outperforming traditional staging systems with higher AUC and C‐index values. The incorporation of TIES into predictive models significantly enhances prognostic accuracy, refining risk stratification and providing a more precise assessment of patient outcomes.

In recent years, PD‐1/PD‐L1 inhibitors have revolutionized gastric cancer treatment, with nivolumab plus chemotherapy now established as a first‐line standard [54]. However, immunotherapy response rates remain below 50%, and aside from microsatellite instability‐high (MSI‐H) status, reliable predictive biomarkers are lacking [3]. The prognostic value of PD‐L1 expression and tumor mutational burden (TMB) remains controversial, underscoring the need for alternative indicators of the tumor immune microenvironment [3]. The TME plays a pivotal role in both patient survival and immunotherapy response. High tumor‐infiltrating lymphocyte (TIL) density correlates with better prognosis, whereas increased FOXP3+ regulatory T cells (Tregs) and M2‐polarized macrophages predict worse outcomes [55]. Incorporating TME assessment into prognostic models could refine treatment decision‐making and patient selection for immunotherapy. In this study, we developed TIES, an immune‐related prognostic model that effectively stratifies patients into high‐ and low‐risk groups. Compared to existing biomarkers, TIES demonstrated superior predictive accuracy for OS and DFS in the PD‐L1‐treated cohort, outperforming TIDE in risk stratification. Notably, the low‐TIES group exhibited a more favorable prognosis, which may be attributed to its reduced infiltration of immunosuppressive cells and enhanced anti‐tumor immune activity. Furthermore, the predictive utility of TIES in immunotherapy was preliminarily validated. In gastric cancer patients treated with immune checkpoint inhibitors, the low‐TIES group showed a significant survival benefit, whereas TIDE failed to distinguish prognostic differences. The lower infiltration of immunosuppressive profile observed in the low‐TIES group may explain its enhanced responsiveness to immunotherapy, reinforcing TIES as a more reliable predictive tool. The reduced infiltration of immunosuppressive cells in the low‐TIES group may account for its heightened responsiveness to immunotherapy, reinforcing TIES as a reliable predictive tool. In conclusion, TIES provides a comprehensive assessment of the immune microenvironment in gastric cancer and serves as a promising biomarker for identifying patients most likely to benefit from immunotherapy. Its implementation in clinical practice could facilitate personalized treatment strategies and ultimately improve patient outcomes.

Clinically, incorporating TIES alongside conventional parameters significantly improved prognostic precision. Decision‐curve and reclassification analyses demonstrated that TIES + stage models yield superior net clinical benefit compared to TNM staging alone, reinforcing its value as an adjunct to existing risk stratification systems. Beyond prognosis, TIES also delineates distinct immunobiological subtypes, offering potential guidance for treatment decisions—particularly in identifying patients likely to benefit from immunotherapy or stroma‐targeted approaches such as TGF‐β blockade or CAF inhibition.

Nevertheless, this study has several limitations. The retrospective design and heterogeneity across transcriptomic platforms, despite rigorous batch correction, may introduce residual bias. The immunotherapy validation cohort was relatively small, necessitating confirmation in larger, prospective clinical trials. Furthermore, spatial heterogeneity within the TME, which may influence local immune interactions, was not captured in the current framework. Future integration of spatial transcriptomics and single‐cell RNA sequencing could further refine TIES's capacity to resolve intratumoral immune complexity. Despite these constraints, the multi‐cohort reproducibility, mechanistic coherence, and qPCR validation of TIES provide compelling evidence for its biological and clinical relevance.

## 5 Conclusions

In summary, this study establishes TIES as a robust, reproducible, and biologically grounded immune‐based prognostic model for gastric cancer. By integrating four key immune–stromal axes, TIES transcends conventional clinicopathological metrics to deliver a more precise reflection of tumor–immune dynamics. High TIES values identify tumors with stromal activation and immune exclusion, corresponding to poor outcomes and limited immunotherapy benefit, whereas low TIES indicates immune‐inflamed tumors with a favorable prognosis and heightened responsiveness to checkpoint blockade. The translationally validated 12‐gene qPCR panel (qTIES) further extends the model's clinical applicability, enabling rapid and cost‐effective implementation in real‐world diagnostic workflows.

Together, these findings highlight TIES as both a prognostic biomarker and a predictive tool for immunotherapy, with the potential to guide precision oncology strategies in gastric cancer. Prospective validation and integration of TIES into clinical decision algorithms could pave the way toward immune‐contexture–driven patient management, ultimately improving therapeutic outcomes and personalizing care for patients with gastric cancer.

## Author Contributions

Xinxing Li and Zhiqian Hu conceptualized and designed the study, revised the manuscript, and oversaw the submission process. Jiexuan Wang performed all data analyses, generated all figures, and drafted the manuscript. Xin Yang and Xuan Dai contributed to data collection and curation. All authors reviewed and approved the final version of the manuscript.

## Funding

This study was supported by Tongji Hospital Clinical Research Project (ITJ (ZD)2104), Shanghai Natural Science Foundation General Program (SKW2030), Tongji Hospital Talent Recruitment, Key Projects (RCQD2102), Shanghai Municipal Health Commission Research Project (WSJ2303), Shanghai 2023 Annual ‘Science and Technology Innovation Action Plan’ Special Project for Medical Innovation Research (SKW2311), Tongji Hospital Talent Program: Ganquan Rising Star (HBRC2104) and Clinical ‘Five New’ Innovation and Development Project (ITJ (ZD)2308). The opinions expressed here are those of the authors and do not necessarily represent the official position of the organizations with which they are affiliated.

## Conflicts of Interest

The authors declare no conflicts of interest.

## Supporting information




**Data S1:** Supporting Information.

**Table S1:** Clinical characteristics of gastric cancer patients.
**Table S2:** Genes involved in the 155 immune contexture‐related signatures in the study.
**Table S3:** Identified signatures correlated with OS in Multi‐Cox of training cohorts.
**Table S4:** Multicollinearity test used by variance inflation factors (VIF) and kappa test for selected signatures in all cohorts.
**Table S5:** The C‐index and AUC values for OS and DFS in the training and test cohorts.
**Table S6:** The AUC and *p*‐values for OS and DFS in cohorts with and without TIES.
**Table S7:** GSEA results of the KEGG pathways in the high‐ and low‐TIES groups in TCGA cohort.
**Table S8:** Clinical Features and Survival Outcomes.
**Table S9:** qPCR Primer Sequences.
**Table S10:** qPCR Ct Values Across Samples.
**Table S11:** qTIES Scores and Survival Outcomes.

## Data Availability

The datasets generated and analyzed during the current study are available in Gene‐Expression Omnibus (GEO) at GSE15459, GSE62254, GSE84433, GSE13861, GSE26899, and GSE26901, and in TCGA at TCGA‐STAD.
