# Supplementary material for: Tumor Immune Contexture Model Predicts Prognosis and Immunotherapy Response in Gastric Cancer
Source: J Gene Med. 2025 Dec 17;27(12):e70065. doi: 10.1002/jgm.70065 (PMC12711383; doi:10.1002/jgm.70065)
Supplement: Supplementary file 1 — Data S1: Supporting Information. [file JGM-27-e70065-s001.pdf]

Supplementary Figure

S1. Batch effects and PCA after correction: Samples from four public cohorts (GSE13861, GSE15459, GSE62254, GSE84433) cluster by cohort pre-correction but mix post-correction, indicating effective batch-effect removal.

S2. Feature stability bubble plot: Cohort-wise regression coefficients and significance (color = sign/magnitude; dot size = −log10(FDR)) show cross-cohort consistency of candidate features.

S3. Stage-only baseline performance: Time-dependent AUC and C-index for OS and DFS across all cohorts show limited discrimination when using pathological stage alone.

S4. Internal validation:
A. Forest plot of univariable/multivariable Cox for DFS;
B–D. Calibration curves for OS/DFS with and without tie handling;
E. Time-dependent AUC comparison (with vs. without ties) shows overall better curves when ties are handled.

S5. Decision curves and LODO external validation:
A–B. Decision curve analysis and baseline stability for OS/DFS;
C–D. External validation across leave-one-dataset-out cohorts;
E–F. Stratified sensitivity analyses using a 3-year risk threshold.

S6. Pathway-level biological associations: Hallmark-based GSEA and Spearman correlation heatmaps between TES and pathways in TCGA-STAD, GSE62254, and GSE84433 reveal significant associations with multiple tumor-related pathways.

S7. Clinical/molecular associations — I: Scatter and grouped boxplots of TES vs. selected clinical or molecular features demonstrate consistent associations across cohorts.

S8. Clinical/molecular associations — II: Extended correlation visualizations (scatter + boxplots) further illustrate relationships between TES and additional features.

S1

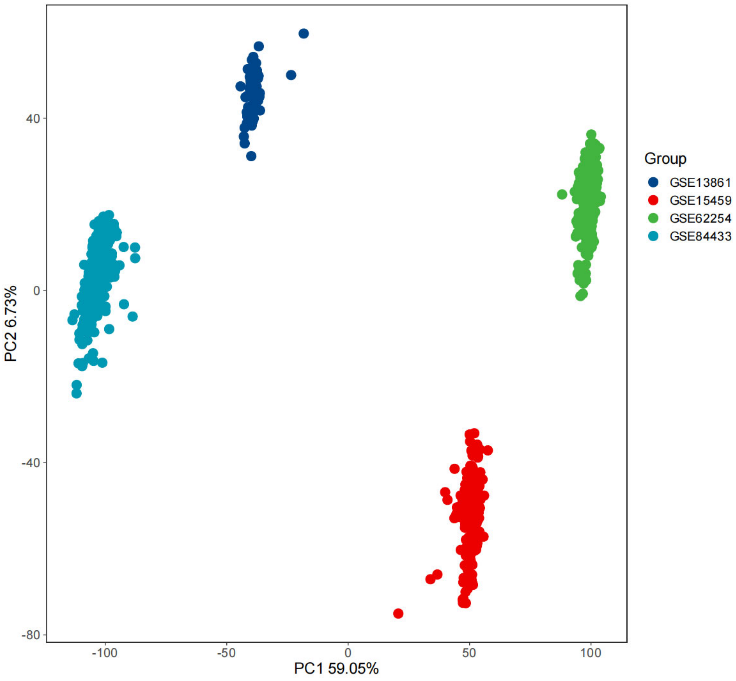

40
0
-40
-80
-100
-50
0
50
100
PC1 59.05%
PC2 6.73%
Group
GSE13861
GSE15459
GSE62254
GSE84433

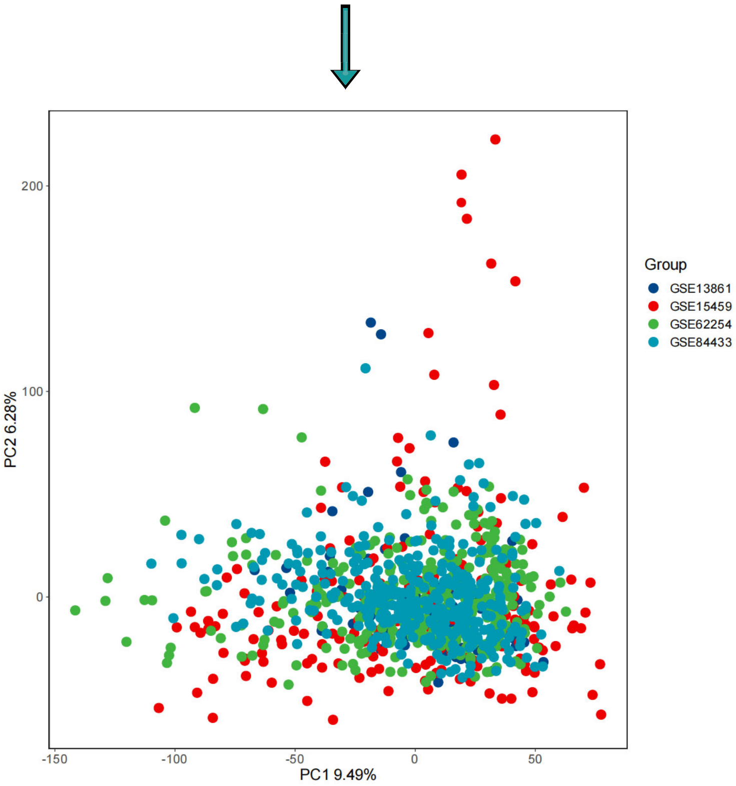

200
100
0
-150
-100
-50
0
50
PC1 9.49%
PC2 6.28%
Group
GSE13861
GSE15459
GSE62254
GSE84433

S2

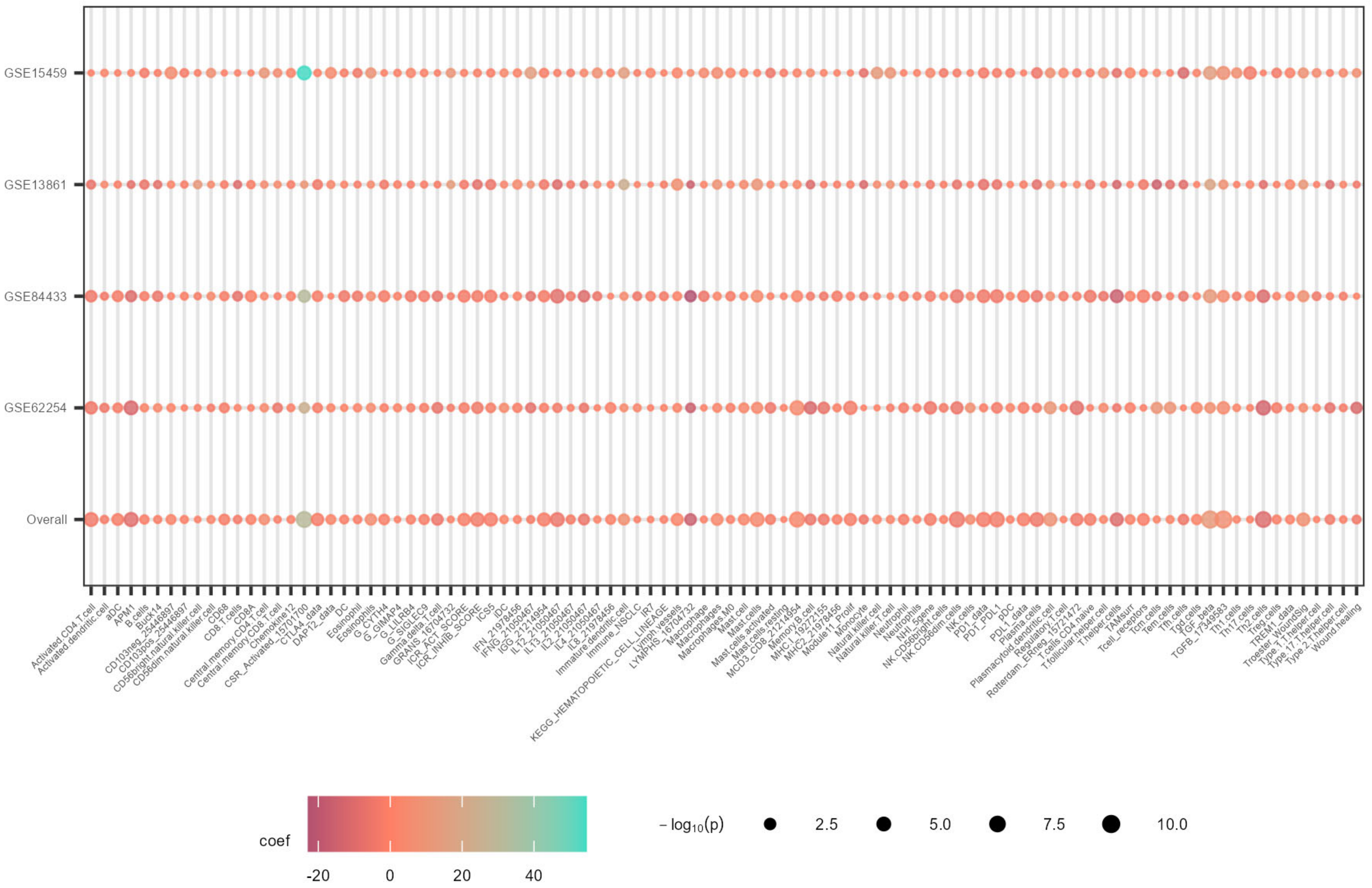
GSE15459
GSE13861
GSE84433
GSE62254
Overall
Activated.CD4.T.cell
Activated.dendritic.cell
aDC
APM1
B.cells
Buck14
CD103neg_25446897
CD103pos_25446897
CD56bright.natural.killer.cell
CD56dim.natural.killer.cell
CD68
CD8.T.cells
CD8A
Central.memory.CD4.T.cell
Central.memory.CD8.T.cell
Chemokine12
CSR_Activated_15701700
CTLA4_data
DAP12_data
DC
Eosinophil
Eosinophils
G_CYTH4
G_GIMAP4
G_LILRB4
G_SIGLEC9
Gamma.delta.T.cell
GRANS_16704732
ICR_ACT_SCORE
ICR_INHIB_SCORE
ICS5
iDC
IFN_21978456
IFNG_21050467
IGG_21214954
IL12_21050467
IL13_21050467
IL2_21050467
IL4_21050467
IL8_21978456
Immature.dendritic.cell
Immune_NSCLC
IR7
KEGG_HEMATOPOIETIC_CELL_LINEAGE
Lymph.vessels
LYMPHS_16704732
Macrophage
Macrophages
Macrophages.M0
Mast.cell
Mast.cells
Mast.cells.activated
Mast.cells.resting
MCD3_CD8_21214954
Memory.B.cell
MHC.I_19272155
MHC2_21978456
Module11_Prolif
Monocyte
Natural.killer.cell
Natural.killer.T.cell
Neutrophil
Neutrophils
NHI_5gene
NK.CD56bright.cells
NK.CD56dim.cells
NK.cells
PD1_data
PD1_PDL1
pDC
PDL1_data
Plasma.cells
Plasmacytoid.dendritic.cell
Regulatory.T.cell
Rotterdam_ERneg_15721472
T.cells.CD4.naive
T.follicular.helper.cell
T.helper.cells
TAMsurr
Tcell_receptors
Tcm.cells
Tem.cells
Tfh.cells
Tgd.cells
TGF_beta
TGFB_17349583
Th1.cells
Th17.cells
Th2.cells
Treg.cells
TREM1_data
Troester_WoundSig
Type.1.T.helper.cell
Type.17.T.helper.cell
Type.2.T.helper.cell
Wound.healing
coef
-20
0
20
40
$-\log_{10}(p)$
2.5
5.0
7.5
10.0

S3

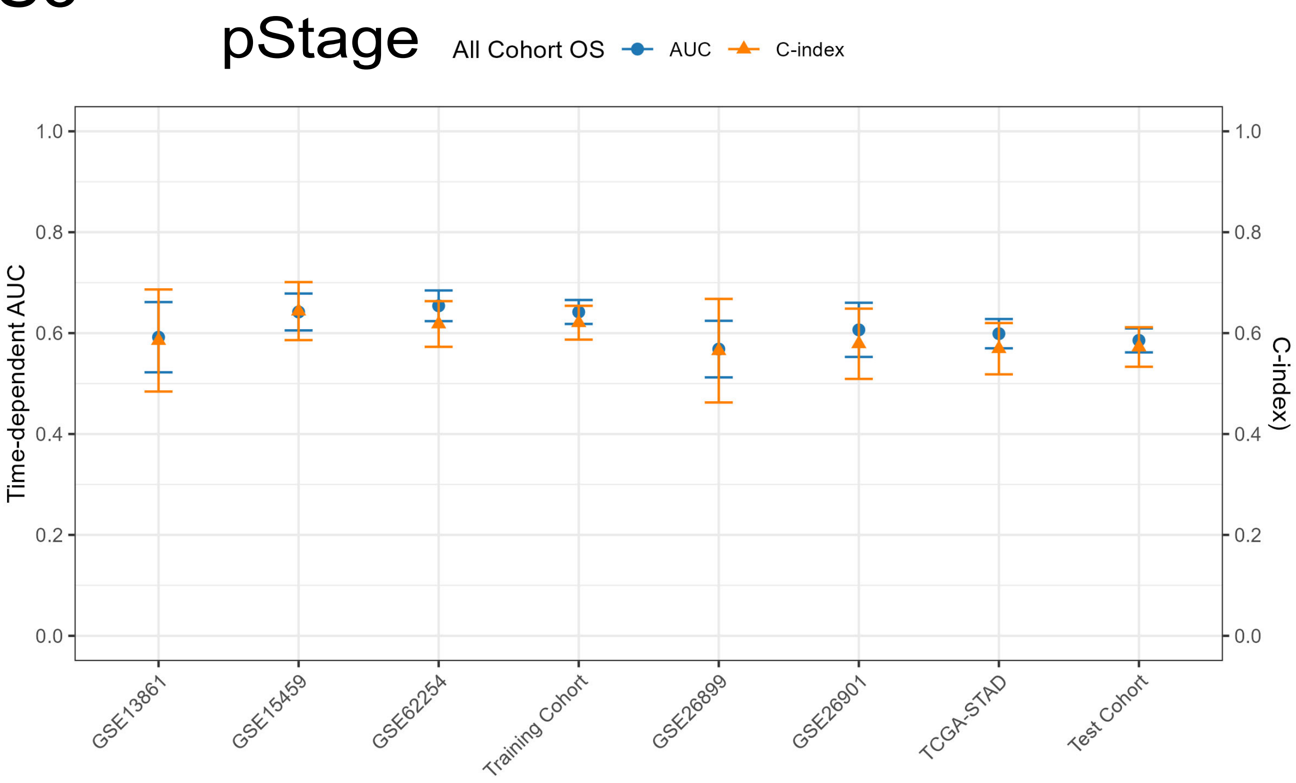
pStage
All Cohort OS
AUC
C-index
Time-dependent AUC
C-index)
1.0
0.8
0.6
0.4
0.2
0.0
GSE13861
GSE15459
GSE62254
Training Cohort
GSE26899
GSE26901
TCGA-STAD
Test Cohort

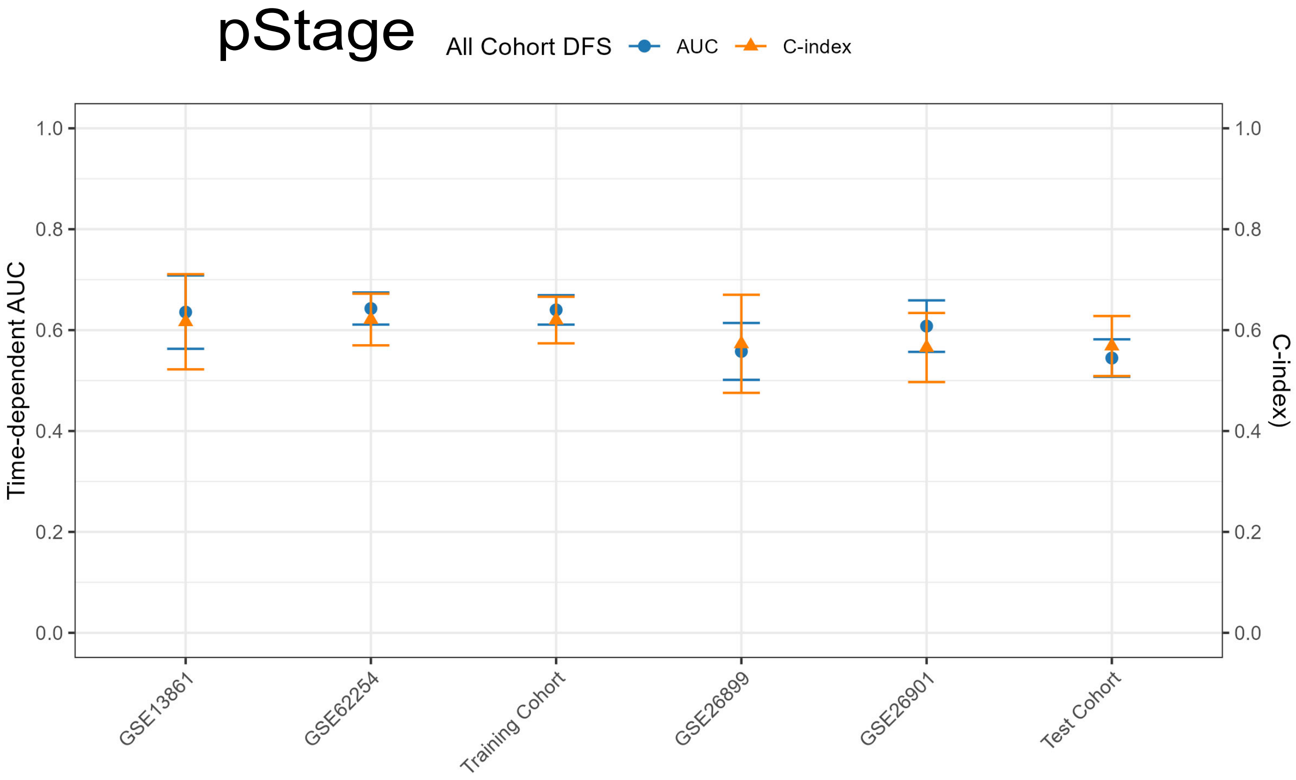
pStage
All Cohort DFS
AUC
C-index
Time-dependent AUC
C-index)
1.0
0.8
0.6
0.4
0.2
0.0
GSE13861
GSE62254
Training Cohort
GSE26899
GSE26901
Test Cohort

S4

A

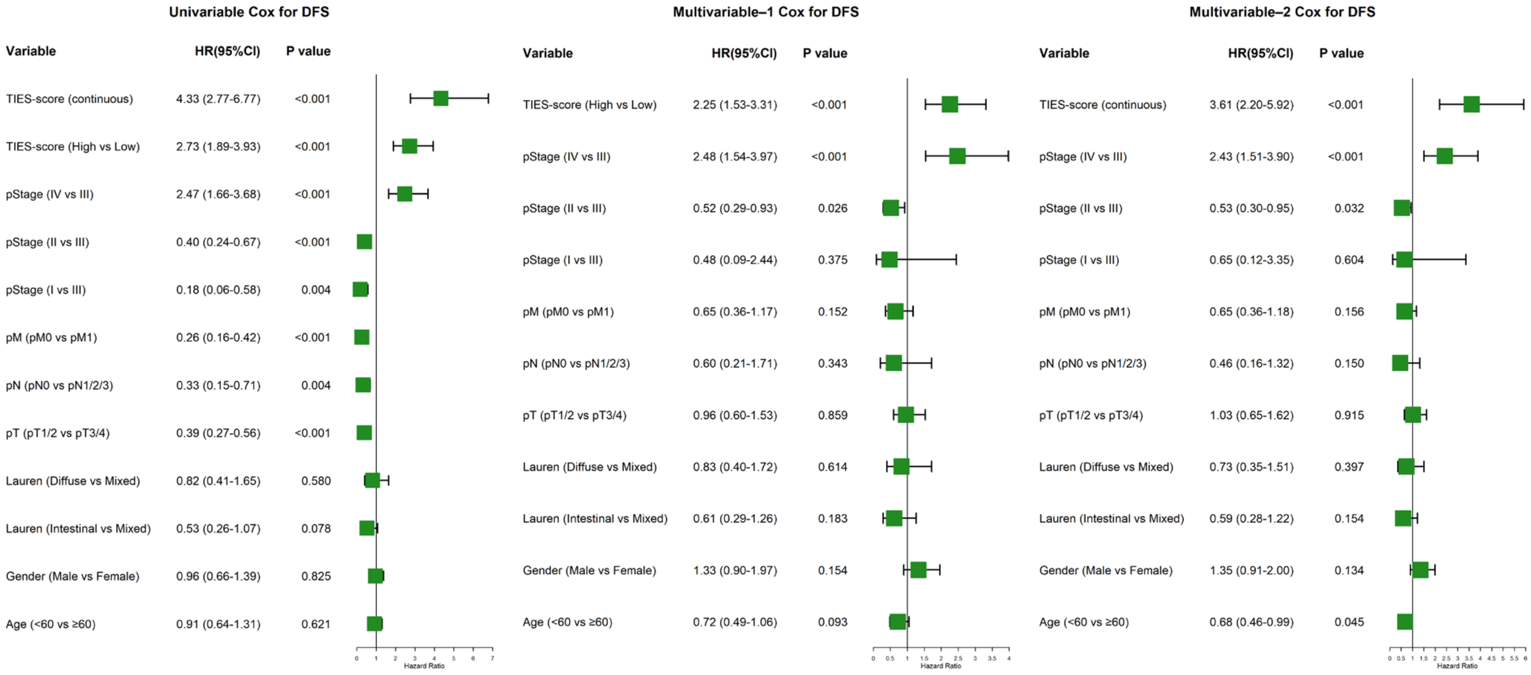

B

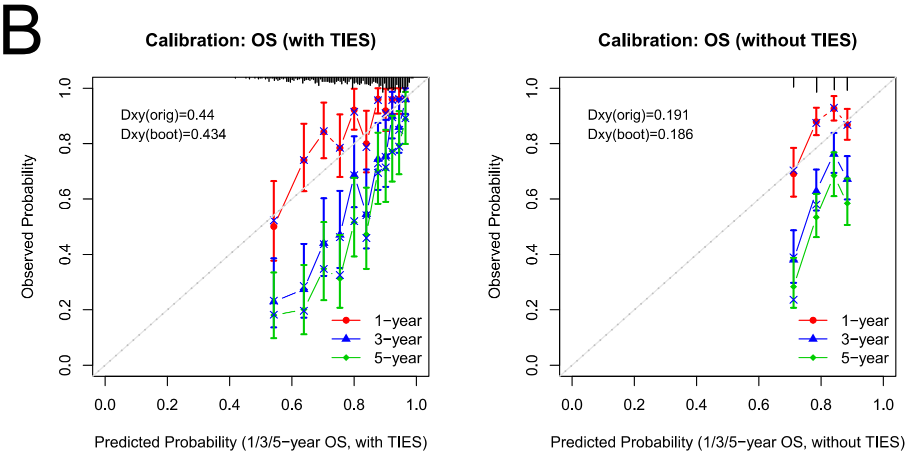

C

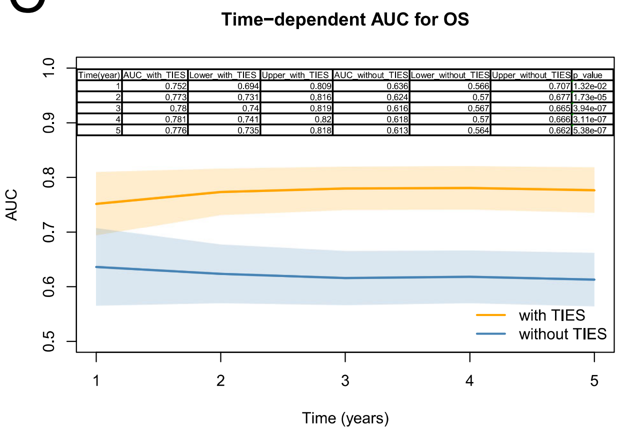

Time(year)	AUC_with_TIES	Lower_with_TIES	Upper_with_TIES	AUC_without_TIES	Lower_without_TIES	Upper_without_TIES	p_value
1	0.752	0.694	0.809	0.636	0.566	0.707	1.32e-02
2	0.773	0.731	0.816	0.624	0.57	0.677	1.73e-05
3	0.78	0.74	0.819	0.616	0.567	0.665	3.94e-07
4	0.781	0.741	0.82	0.618	0.57	0.666	3.11e-07
5	0.776	0.735	0.818	0.613	0.564	0.662	5.38e-07

E

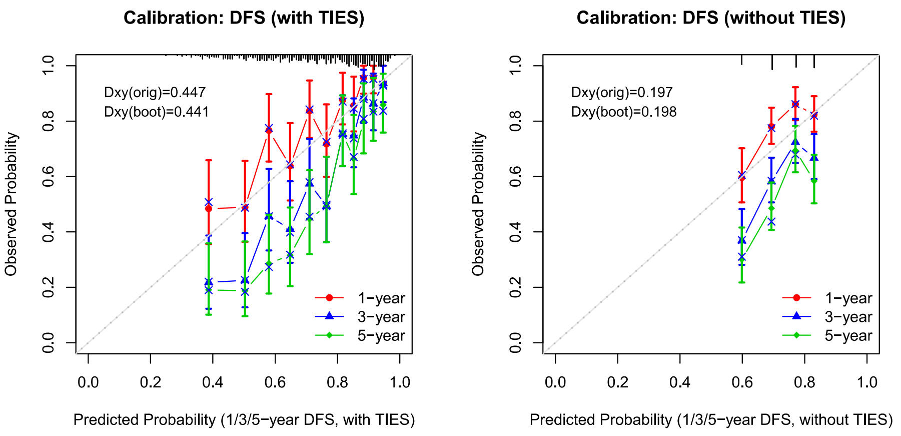

F

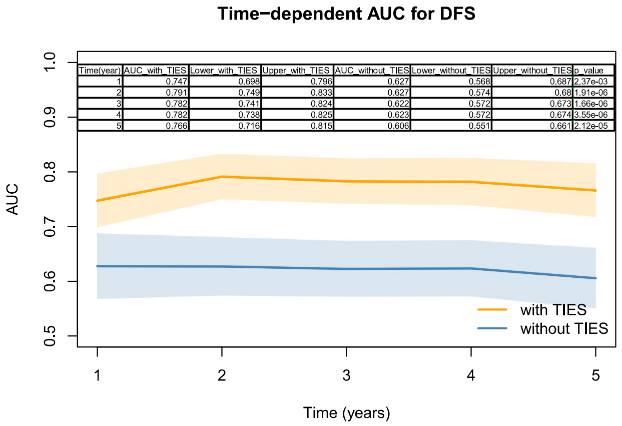

Time(year)	AUC_with_TIES	Lower_with_TIES	Upper_with_TIES	AUC_without_TIES	Lower_without_TIES	Upper_without_TIES	p_value
1	0.747	0.698	0.796	0.627	0.568	0.687	2.37e-03
2	0.791	0.749	0.833	0.627	0.574	0.68	1.91e-06
3	0.782	0.741	0.824	0.622	0.572	0.673	1.66e-06
4	0.782	0.738	0.825	0.623	0.572	0.674	3.55e-06
5	0.766	0.716	0.815	0.606	0.551	0.661	2.12e-05

S5

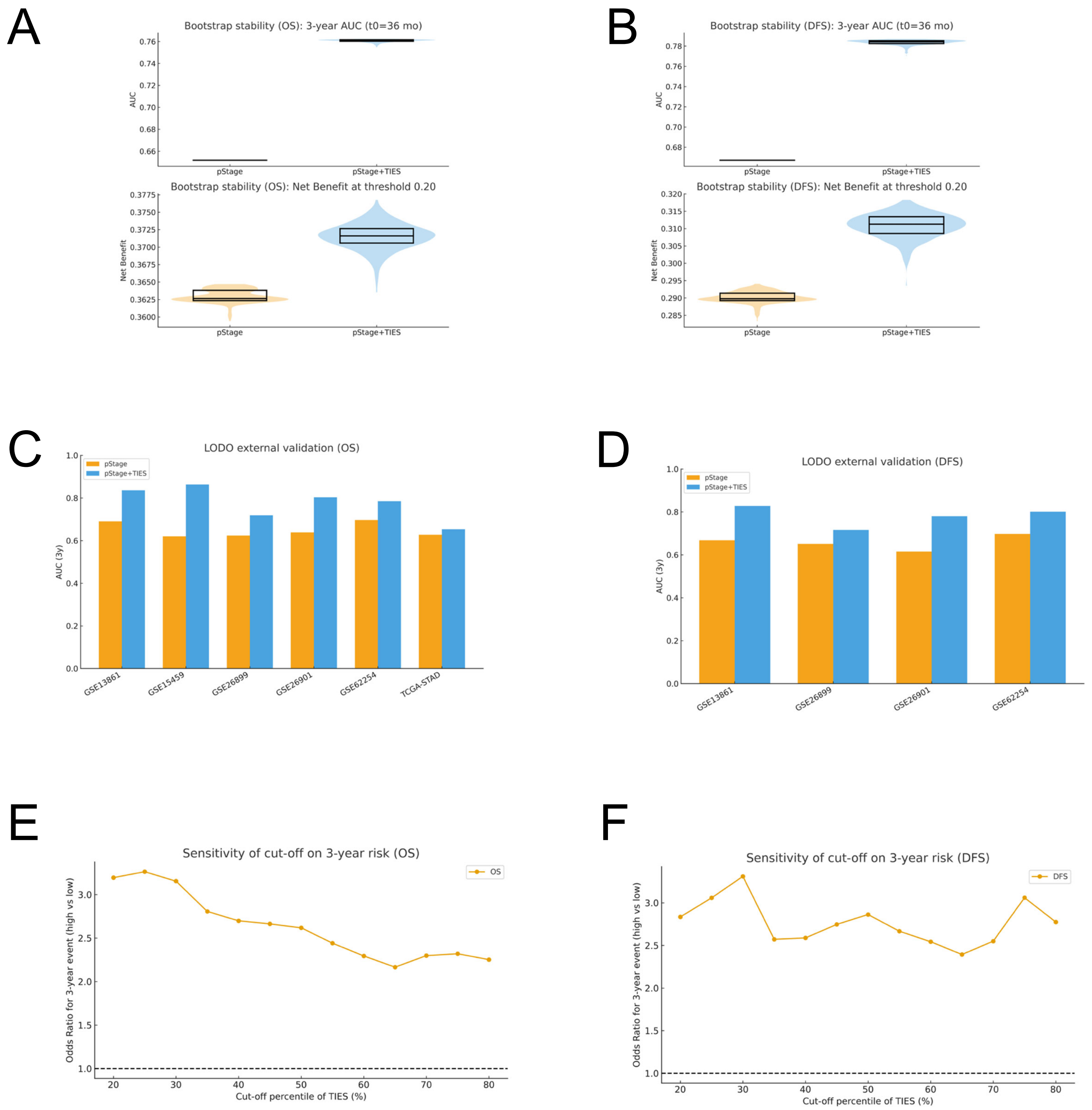
A
Bootstrap stability (OS): 3-year AUC (t0=36 mo)
AUC
0.76
0.74
0.72
0.70
0.68
0.66
pStage
pStage+TIES
Bootstrap stability (OS): Net Benefit at threshold 0.20
Net Benefit
0.3775
0.3750
0.3725
0.3700
0.3675
0.3650
0.3625
0.3600
pStage
pStage+TIES
B
Bootstrap stability (DFS): 3-year AUC (t0=36 mo)
AUC
0.78
0.76
0.74
0.72
0.70
0.68
pStage
pStage+TIES
Bootstrap stability (DFS): Net Benefit at threshold 0.20
Net Benefit
0.320
0.315
0.310
0.305
0.300
0.295
0.290
0.285
pStage
pStage+TIES
C
LODO external validation (OS)
AUC (3y)
pStage
pStage+TIES
1.0
0.8
0.6
0.4
0.2
0.0
GSE13861
GSE15459
GSE26899
GSE26901
GSE62254
TCGA-STAD
D
LODO external validation (DFS)
AUC (3y)
pStage
pStage+TIES
1.0
0.8
0.6
0.4
0.2
0.0
GSE13861
GSE26899
GSE26901
GSE62254
E
Sensitivity of cut-off on 3-year risk (OS)
OS
Odds Ratio for 3-year event (high vs low)
3.0
2.5
2.0
1.5
1.0
20
30
40
50
60
70
80
Cut-off percentile of TIES (%)
F
Sensitivity of cut-off on 3-year risk (DFS)
DFS
Odds Ratio for 3-year event (high vs low)
3.0
2.5
2.0
1.5
1.0
20
30
40
50
60
70
80
Cut-off percentile of TIES (%)

S6

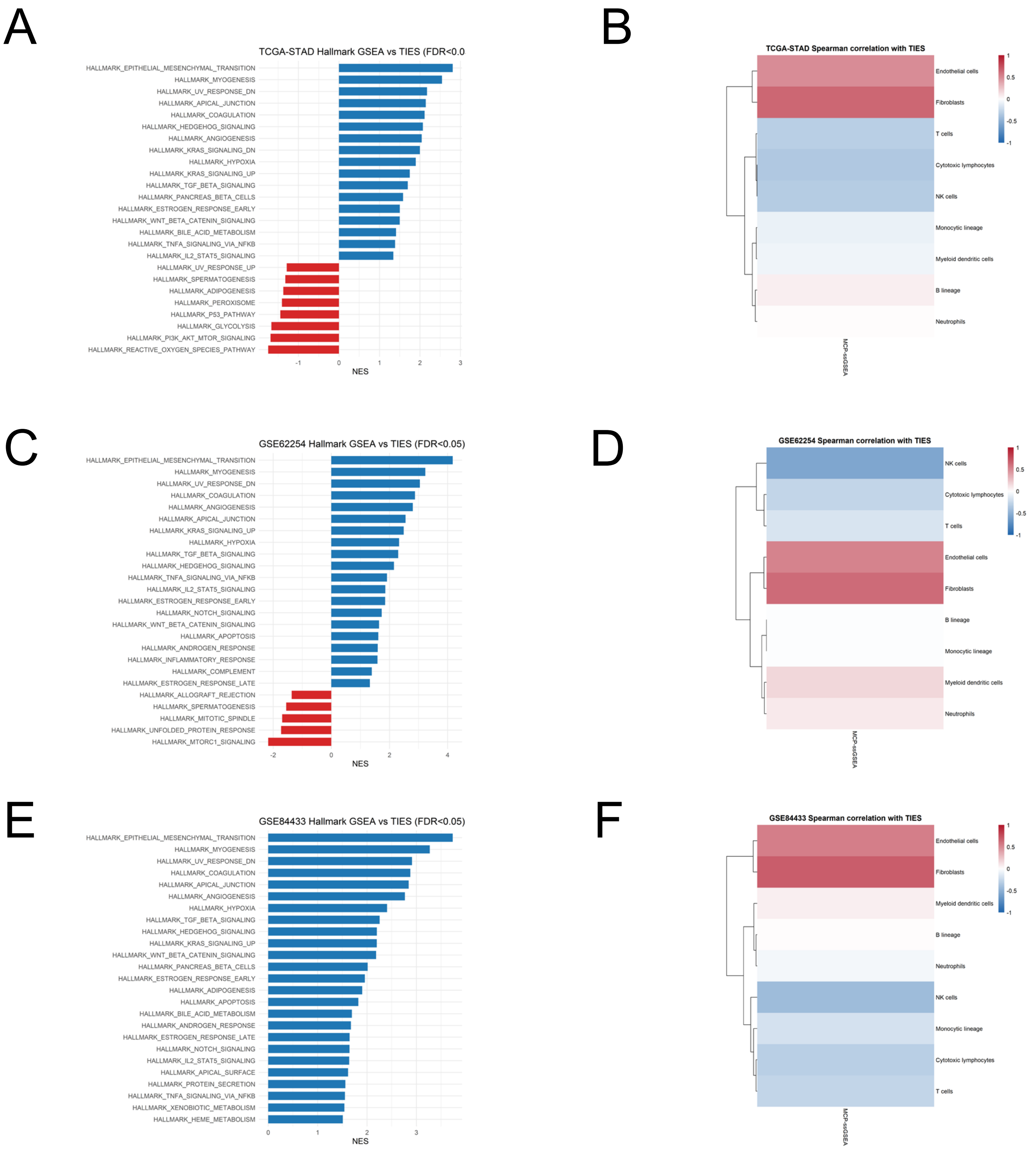
A
TCGA-STAD Hallmark GSEA vs TIES (FDR<0.0
HALLMARK_EPITHELIAL_MESENCHYMAL_TRANSITION
HALLMARK_MYOGENESIS
HALLMARK_UV_RESPONSE_DN
HALLMARK_APICAL_JUNCTION
HALLMARK_COAGULATION
HALLMARK_HEDGEHOG_SIGNALING
HALLMARK_ANGIOGENESIS
HALLMARK_KRAS_SIGNALING_DN
HALLMARK_HYPOXIA
HALLMARK_KRAS_SIGNALING_UP
HALLMARK_TGF_BETA_SIGNALING
HALLMARK_PANCREAS_BETA_CELLS
HALLMARK_ESTROGEN_RESPONSE_EARLY
HALLMARK_WNT_BETA_CATENIN_SIGNALING
HALLMARK_BILE_ACID_METABOLISM
HALLMARK_TNFA_SIGNALING_VIA_NFKB
HALLMARK_IL2_STAT5_SIGNALING
HALLMARK_UV_RESPONSE_UP
HALLMARK_SPERMATOGENESIS
HALLMARK_ADIPOGENESIS
HALLMARK_PEROXISOME
HALLMARK_P53_PATHWAY
HALLMARK_GLYCOLYSIS
HALLMARK_PI3K_AKT_MTOR_SIGNALING
HALLMARK_REACTIVE_OXYGEN_SPECIES_PATHWAY
-1
0
1
2
3
NES
B
TCGA-STAD Spearman correlation with TIES
Endothelial cells
Fibroblasts
T cells
Cytotoxic lymphocytes
NK cells
Monocytic lineage
Myeloid dendritic cells
B lineage
Neutrophils
MCP-ssGSEA
1
0.5
0
-0.5
-1
C
GSE62254 Hallmark GSEA vs TIES (FDR<0.05)
HALLMARK_EPITHELIAL_MESENCHYMAL_TRANSITION
HALLMARK_MYOGENESIS
HALLMARK_UV_RESPONSE_DN
HALLMARK_COAGULATION
HALLMARK_ANGIOGENESIS
HALLMARK_APICAL_JUNCTION
HALLMARK_KRAS_SIGNALING_UP
HALLMARK_HYPOXIA
HALLMARK_TGF_BETA_SIGNALING
HALLMARK_HEDGEHOG_SIGNALING
HALLMARK_TNFA_SIGNALING_VIA_NFKB
HALLMARK_IL2_STAT5_SIGNALING
HALLMARK_ESTROGEN_RESPONSE_EARLY
HALLMARK_NOTCH_SIGNALING
HALLMARK_WNT_BETA_CATENIN_SIGNALING
HALLMARK_APOPTOSIS
HALLMARK_ANDROGEN_RESPONSE
HALLMARK_INFLAMMATORY_RESPONSE
HALLMARK_COMPLEMENT
HALLMARK_ESTROGEN_RESPONSE_LATE
HALLMARK_ALLOGRAFT_REJECTION
HALLMARK_SPERMATOGENESIS
HALLMARK_MITOTIC_SPINDLE
HALLMARK_UNFOLDED_PROTEIN_RESPONSE
HALLMARK_MTORC1_SIGNALING
-2
0
2
4
NES
D
GSE62254 Spearman correlation with TIES
NK cells
Cytotoxic lymphocytes
T cells
Endothelial cells
Fibroblasts
B lineage
Monocytic lineage
Myeloid dendritic cells
Neutrophils
MCP-ssGSEA
1
0.5
0
-0.5
-1
E
GSE84433 Hallmark GSEA vs TIES (FDR<0.05)
HALLMARK_EPITHELIAL_MESENCHYMAL_TRANSITION
HALLMARK_MYOGENESIS
HALLMARK_UV_RESPONSE_DN
HALLMARK_COAGULATION
HALLMARK_APICAL_JUNCTION
HALLMARK_ANGIOGENESIS
HALLMARK_HYPOXIA
HALLMARK_TGF_BETA_SIGNALING
HALLMARK_HEDGEHOG_SIGNALING
HALLMARK_KRAS_SIGNALING_UP
HALLMARK_WNT_BETA_CATENIN_SIGNALING
HALLMARK_PANCREAS_BETA_CELLS
HALLMARK_ESTROGEN_RESPONSE_EARLY
HALLMARK_ADIPOGENESIS
HALLMARK_APOPTOSIS
HALLMARK_BILE_ACID_METABOLISM
HALLMARK_ANDROGEN_RESPONSE
HALLMARK_ESTROGEN_RESPONSE_LATE
HALLMARK_NOTCH_SIGNALING
HALLMARK_IL2_STAT5_SIGNALING
HALLMARK_APICAL_SURFACE
HALLMARK_PROTEIN_SECRETION
HALLMARK_TNFA_SIGNALING_VIA_NFKB
HALLMARK_XENOBIOTIC_METABOLISM
HALLMARK_HEME_METABOLISM
0
1
2
3
NES
F
GSE84433 Spearman correlation with TIES
Endothelial cells
Fibroblasts
Myeloid dendritic cells
B lineage
Neutrophils
NK cells
Monocytic lineage
Cytotoxic lymphocytes
T cells
MCP-ssGSEA
1
0.5
0
-0.5
-1

S7

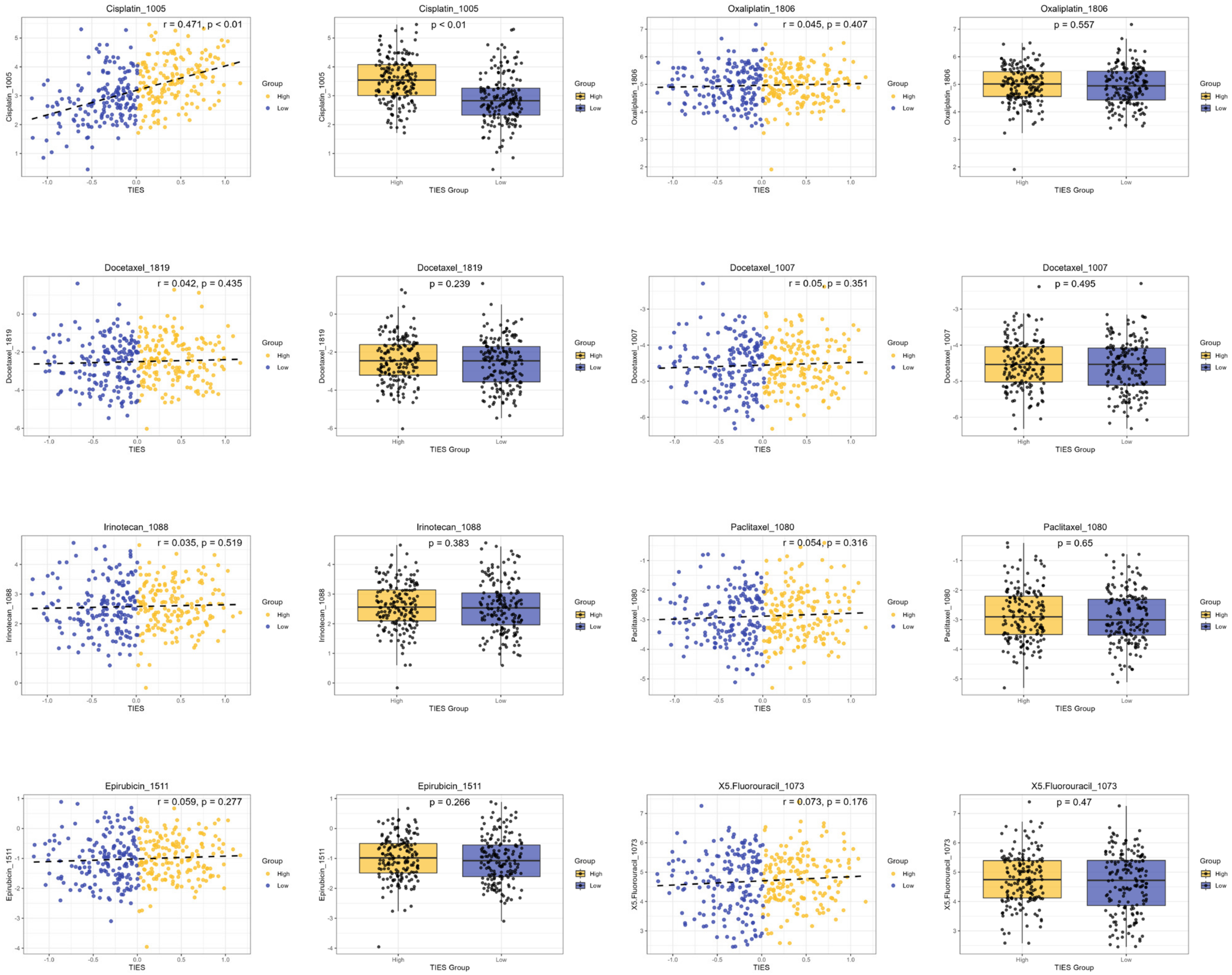

Cisplatin_1005
r = 0.471, p < 0.01
Cisplatin_1005
TIES
Group
High
Low
Cisplatin_1005
p < 0.01
Cisplatin_1005
TIES Group
Oxaliplatin_1806
r = 0.045, p = 0.407
Oxaliplatin_1806
TIES
Oxaliplatin_1806
p = 0.557
Oxaliplatin_1806
TIES Group
Docetaxel_1819
r = 0.042, p = 0.435
Docetaxel_1819
TIES
Docetaxel_1819
p = 0.239
Docetaxel_1819
TIES Group
Docetaxel_1007
r = 0.05, p = 0.351
Docetaxel_1007
TIES
Docetaxel_1007
p = 0.495
Docetaxel_1007
TIES Group
Irinotecan_1088
r = 0.035, p = 0.519
Irinotecan_1088
TIES
Irinotecan_1088
p = 0.383
Irinotecan_1088
TIES Group
Paclitaxel_1080
r = 0.054, p = 0.316
Paclitaxel_1080
TIES
Paclitaxel_1080
p = 0.65
Paclitaxel_1080
TIES Group
Epirubicin_1511
r = 0.059, p = 0.277
Epirubicin_1511
TIES
Epirubicin_1511
p = 0.266
Epirubicin_1511
TIES Group
X5.Fluorouracil_1073
r = 0.073, p = 0.176
X5.Fluorouracil_1073
TIES
X5.Fluorouracil_1073
p = 0.47
X5.Fluorouracil_1073
TIES Group

S8

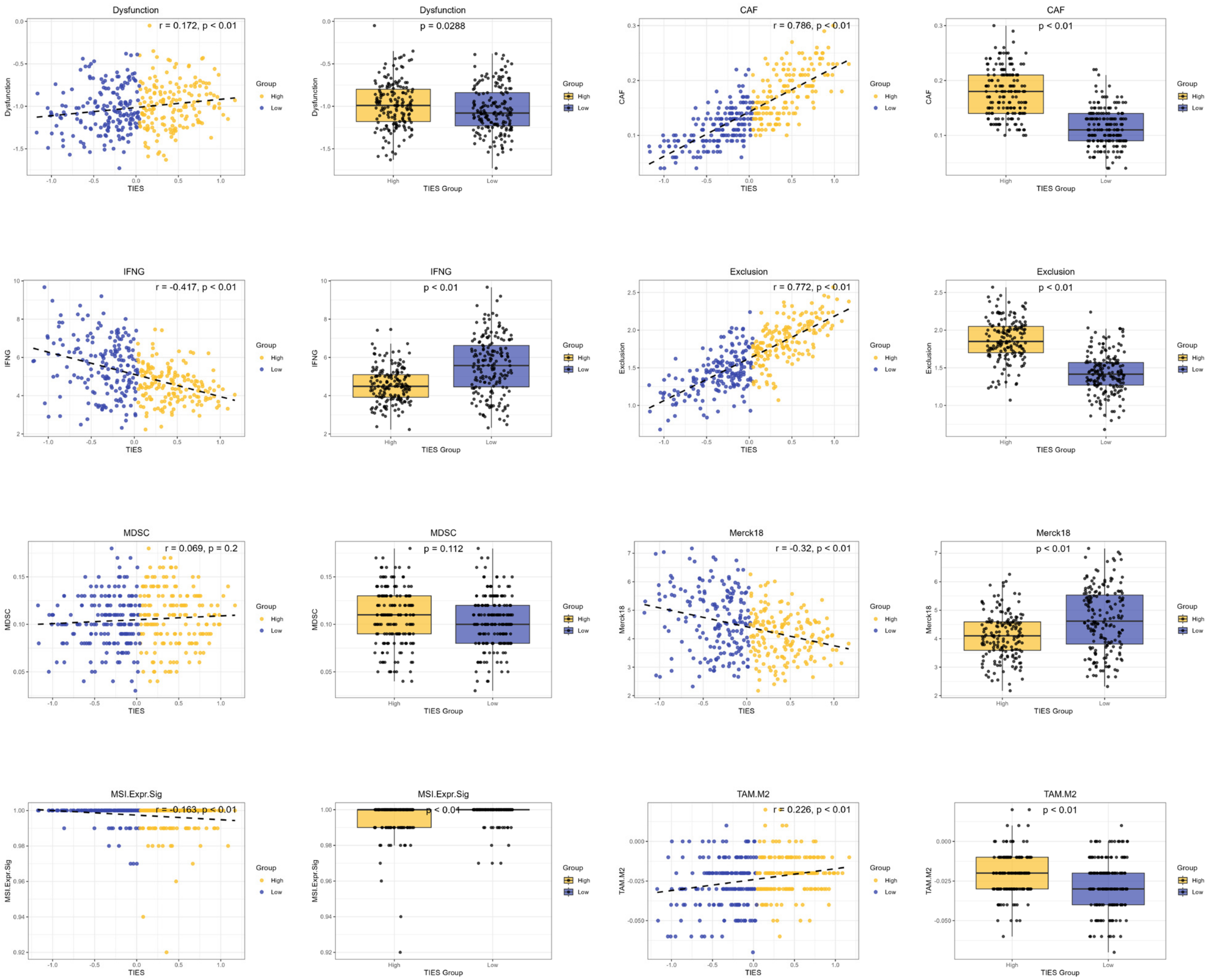
Dysfunction
r = 0.172, p < 0.01
Dysfunction
p = 0.0288
CAF
r = 0.786, p < 0.01
CAF
p < 0.01
IFNG
r = -0.417, p < 0.01
IFNG
p < 0.01
Exclusion
r = 0.772, p < 0.01
Exclusion
p < 0.01
MDSC
r = 0.069, p = 0.2
MDSC
p = 0.112
Merck18
r = -0.32, p < 0.01
Merck18
p < 0.01
MSI.Expr.Sig
r = -0.163, p < 0.01
MSI.Expr.Sig
p < 0.01
TAM.M2
r = 0.226, p < 0.01
TAM.M2
p < 0.01
TIES
TIES Group
High
Low
Group
